# The super-enhancer-driven lncRNA LINC00880 acts as a scaffold between CDK1 and PRDX1 to sustain the malignance of lung adenocarcinoma

**DOI:** 10.1038/s41419-023-06047-w

**Published:** 2023-08-24

**Authors:** Yipeng Feng, Te Zhang, Zeyu Zhang, Yingkuang Liang, Hui Wang, Yuzhong Chen, Xinnian Yu, Xuming Song, Qixing Mao, Wenjie Xia, Bing Chen, Lin Xu, Gaochao Dong, Feng Jiang

**Affiliations:** 1 Department of Thoracic Surgery, Nanjing Medical University Affiliated Cancer Hospital & Jiangsu Cancer Hospital & Jiangsu Institute of Cancer Research, 21009 Nanjing, China; 2 Jiangsu Key Laboratory of Molecular and Translational Cancer Research, Cancer Institute of Jiangsu Province, Nanjing, China; 3 The Fourth Clinical College of Nanjing Medical University, Nanjing, China; 4 Collaborative Innovation Center for Cancer Personalized Medicine, Nanjing Medical University, Nanjing, China

**Keywords:** Cancer epidemiology, Cancer epidemiology

## Abstract

Super-enhancers (SEs) are regulatory element clusters related to cell identity and disease. While the studies illustrating the function of SE-associated long noncoding RNAs (lncRNAs) in lung adenocarcinoma (LUAD) remains few. In our research, a SE-driven lncRNA, LINC00880, was identified, which showed higher expression in LUAD compared to normal tissues and indicated worse outcomes in stage I LUADs. We found that the transcription factor (TF) FOXP3 could simultaneously occupy the promoter and SE regions of LINC00880 to promote its transcription. The oncogenic function of LINC00880 was validated both in vitro and in vivo. Mechanically, LINC00880 binds to the protein CDK1 to increase its kinase activity, which rely on the phosphorylation state of pT161 in CDK1. LINC00880 also promotes the interaction between CDK1 and PRDX1. Moreover, LINC00880 interacts with PRDX1, which indicates that LINC00880 acts as a protein scaffold between CDK1 and PRDX1 to form a ternary complex, thereby resulting in the activation of PI3K/AKT to promote malignancy. Our results reveal that the SE-associated lncRNA LINC00880 regulates the CDK1/PRDX1 axis to sustain the malignancy of LUAD, providing a novel therapeutic target.

## Highlights

- LINC00880 is driven by Super-enhancer.
- LINC00880 interacts with CDK1 to activate its kinase activity.
- LINC00880 acts as the protein scaffold between CDK1 and PRDX1.

## Introduction

The primary reason for cancer-related deaths globally is lung cancer, and its most common pathological subtype is LUAD, and more than 40% of lung cancer fatalities are caused by LUAD [1]. With the emergence of immune checkpoint inhibitors (ICIs) and targeted tyrosine kinase inhibitors (TKIs), the survival rate of LUAD patients has significantly improved, but its overall survival rate remains unsatisfactorily low [2]. An accurate understanding of the pathophysiological mechanisms of LUAD is still lacking.

SEs are defined as clusters of regulatory elements that possess a high degree of either histone H3 lysine 27 acetylation (H3K27ac) or lysine 4 mono-methylation (H3K4me1) [3] and are characterized by the significant enrichment of mediators, chromatin factors, coactivators, and master regulators of cells [4], thereby resulting in high-level gene expression to determine cell identity and coordinate disease mechanisms [5]. Our group brought attention to the multiple functions of SEs in LUAD. We previously identified that activated SEs in LUAD recruit 3 master transcription factors (TFs); including ELF3, EHF, and TGIF1; to form the core transcriptional regulatory circuitry (CRC) [6]. In a recent study, we also discovered that a long noncoding RNA (lncRNA), named LINC01977, hijacked by SEs, promoted LUAD malignancy through the typical TGF-β/SMAD3 pathway [7]. Characterizing such SE-associated lncRNAs could open new avenues to identify targets to halt cancer progression [8, 9], while the mechanisms and functions of these lncRNAs in LUAD remain elusive.

Cyclin-dependent kinases (CDKs) are serine/threonine kinases that drive major cell cycle events in eukaryotic cells [10]. Among them, CDK1 is essential to accelerate cell cycle from phase G1/S to G2/M [11]. Over the past decade, it has been shown that CDK1 interacts with different substrate proteins to perform multiple biological functions beyond cell cycle regulation, including transcriptional regulation, DNA damage repair, epigenetic regulation, and metabolism [12]. A recent study reported that tumor initiation in human melanoma was promoted by CDK1-mediated SOX2 interactions [13]. Little is known about whether CDK1 can interact with lncRNAs to modulate its function. In present study, we identified a SE-associated lncRNA known as LINC00880, which was regulated by FOXP3 through the SE of LINC00880. We further showed that LINC00880 promoted LUAD progression both in vitro and in vivo by scaffolding CDK1 and PRDX1 to form a complex, thereby regulating the PTEN/AKT pathway. It was also found that higher LINC00880 expression indicated poor prognosis in early-stage LUAD patients, suggesting that LINC00880 might be a promising prognostic indicator.

## Methods

### Tissue specimens’ acquisition

At the Nanjing Medical University Affiliated Cancer Hospital, we obtained LUAD samples and matched neighboring normal tissues from patients. All samples were acquired from patients who had LUAD resection and were evaluated by expert pathologists at Nanjing Medical University Affiliated Cancer Hospital. All samples were taken from the biobank of Nanjing Medical University Affiliated Cancer Hospital. All patients completed an informed consent form before donating samples.

Patients’ tissues were acquired, liquid nitrogen was instantly used to freeze tissue, and then kept until usage at −80 °C. For the SE-associated lncRNA microarray, 5 matched LUAD samples and neighboring normal samples were employed [7]. 40 LUAD sample and neighboring normal samples were used to extract RNA for qRT-PCR. 152 LUAD tumor tissue samples were used to extract RNA for qRT-PCR and overnight formalin fixation, followed by embedding in paraffin by a regular protocol. All participants signed written informed consent. The ethical committee of Jiangsu Cancer Hospital Nanjing, China, accepted the research protocol. Supplementary Table 1 contains all clinicopathological data.

### Cell lines and cell culture

The LUAD cell lines (A549, A427, H1299, H1975, H358, PC9) and the human bronchial epithelial cell line (HBE) were obtained from the Shanghai Institute of Cell Biology cell bank (Chinese Academy of Medical Science, Shanghai, China). Based on short tandem repeat (STR) profiling by the manufacturers, none of the cell lines utilized in this research were present in the database of commonly misidentified cell lines. At 37 °C and 5% CO_2,_ RPMI-1640 medium (Gibco, USA) or Dulbecco’s modified Eagle’s medium (DMEM, Gibco) supplemented with 10% fetal bovine serum (FBS, Corning) and 1% penicillin-streptomycin (Gibco) were used to maintain LUAD cell lines.

### Cell transfection

Cells were transfected with SiRNAs (RiboBio, Guangzhou, China) by using Lipofectamine RNAiMAX (Thermo, USA) transfection reagent with OptiMEM (Thermo, USA). Lipofectamine 3000 (Thermo, USA) was used to transfect plasmids into cells. Transfections were performed according to manufacturer. After transfection, cells were collected 48 and 72 h later. Supplementary Table 2 list the all the siRNAs sequences used in this study.

### Quantitative real-time PCR (qRT-PCR)

Extraction of total RNA was exerted using TRIzol reagent (Thermo, USA) in accordance with the manufacturer’s protocol. All RNA samples were kept at −80 °C until reverse transcription and qRT-PCR. Using the PrimeScript® RT Reagent Kit (Takara, Japan), cDNA was obtained by RNA reverse transcription. Random hexamers or oligo(dT)18 primers (Qiagen) were used for reverse transcription. According to the manufacturer’s protocol, SYBR Premix ExTaq real-time PCR Kit (Takara, Japan) was used to perform qRT-PCR. Normalization was performed using ACTIN and GAPDH as internal reference controls. 2^-ΔΔCt^ was utilized to count the expression levels of lncRNA and mRNA. Supplementary Table 2 list all the primers information.

### Western blotting

To extract total protein from cells, RIPA lysis buffer supplemented with phosphatase inhibitors was used. Pierce BCA Protein Assay Kit (Thermo, USA) was utilized to standardize the protein concentrations. Proteins were loaded onto 10% SDS-PAGE gels and then transferred to polyvinylidene fluoride membranes. The membranes were then blocked in 5% BSA for 2 h, followed by incubation with the primary antibody overnight at 4 °C, and then incubated with the corresponding secondary antibodies. Grayscale ratio was employed to detect target protein expression by Odyssey Clx Imaging System (LI-COR, USA). Supplementary Table 3 provides all antibody information.

### Subcellular fractionation

Following the manufacturer’s instructions, LINC00880 expression in the cytoplasmic and nuclear fractions were determined by Ambion PARIS Kit (Invitrogen, USA) for the extraction of RNA and protein. qRT-PCR was performed using RNA isolated from the fractions of cytoplasmic and nuclear. Marker of the cytoplasmic was GAPDH, and the marker of nuclear was U6. Supplementary Table 2 list all primer information.

### Fluorescence in situ hybridization (FISH) assay

The lncRNA FISH Kit (RiboBio, Guangzhou, China) was used for the FISH assay. In a nutshell, 0.5% Triton X-100 in PBS was used to fix and permeabilize the cells. The probes of FISH were constructed by RiboBio. Then, hybridization was carried out overnight at 37 °C in the dark in a humidified chamber. LSM710 confocal microscope (Carl Zeiss, Germany) was utilized to capture images. The signals were detected using the 4’,6-diamidino-2-phenylindole (DAPI) and Cy3 channels.

### Rapid amplification of cDNA ends (RACE)

First-strand cDNA synthesis was performed utilizing the total RNA obtained from PC9. According to the manufacturer’s protocol, 5′- and 3′-RACE analysis was carried out utilizing the GeneRacer^TM^ Kit (Thermo, USA). Supplementary Table 2 list the gene-specific primers used for RACE.

### CCK-8 assay

Transfected PC9 and A549 cells were planted into 96-well plates. According to the manufacturer’s protocol, Cell Counting Kit-8 (CCK-8) (Beyotime Biotechnology, Shanghai, China) was utilized to examine the cell viability. Microplate reader (Bio-Rad, Hercules, USA) was utilized to measure the absorbance of cells at 450 nm.

### Real‐time cell analysis (RTCA)

Using cell proliferation plates, cell growth was monitored by the RTCA system. Cells (15,000) were plated into each well of the e-plate after RPMI-1640 or DMEM with 10% FBS was added to the chamber at 37 °C and 5% CO2. Until the experiment ended (up to 50 h), readings were taken every 15 min.

### EdU assay

According to the manufacturer’s protocol, the EdU DNA Proliferation Kit (KeyGene, China) was utilized to measure cell proliferation. A549 and PC9 cells were grown in 96-well plates in full medium until 80% confluent, then treated with 50 μM EdU for 6 hours before being analyzed.

### Colony formation assay

Transfected PC9 and A549 cells were planted into 6-well plates (200 cells/well) to form colonies over 14 days. After the incubation, 4% paraformaldehyde was used to fix cells and 1% crystal violet solution was utilized to stain cells. PBS was used to clean the plates and they were left to dry at ambient temperature. Finally, the colonies number were calculated.

### Transwell migration and invasion test

Transwell chambers (Corning, USA) was utilized to carry outcell invasion test. Each upper chamber was filled with Transfected cells (4 × 10^4^) in 200 μL of 1640/DMEM without serum, while the lower chamber received 500 μL of 1640/DMEM containing 10% FBS. After incubating the cells for 24–48 h, 4% paraformaldehyde was utilized to fix the invading cells in the lower chamber for thirty minutes and then 1% crystal violet was utilized to stain the invading cells for another thirty minutes. Finally, the cells were imaged and counted.

### Wound healing assay

Transfected PC9 and A549 cells were planted into 6-well plates. When the cells had reached 80–90% confluence, a pipette tip was utilized to scratch a straight line in one direction. Then, any detached cells from the cell monolayer were removed by gently washing. This was followed by incubating the remaining cells with serum-free 1640/DMEM for 24–48 h. After the incubation, images of the healing scratch wounds were acquired and analyzed.

### Zebrafish xenograft model and lung metastasis models

Transfected PC9 cells were grown to 90% confluency before being trypsinized, counted, resuspended, and fluorescently labeled. Then, the recipient fish’s peritoneal cavity was injected with 300 fluorescently tagged PC9 cells. Four days after injection, a fluorescence microscope (Leica, Germany) was used to capture the image of the recipient fish. Cell proliferation was evaluated by quantifying the image area of the resulting fish tumors using Image J and then multiplying it by the average fluorescence intensity. Cell invasion ability was determined by quantifying the fluorescence area in fish tails.

We obtained 4- to 6-week-old female BALB/c mice from Gem Pharmatech (China) and maintained them in accordance with the Institutional Animal Care and Use Committee (IACUC) of Nanjing Medical University (IACUC-2012002-1). There was no deliberate exclusion or inclusion of any animals during the sorting procedure. We randomly split 20 female BALB/c mice into four groups. To establish the lung metastasis model, we injected the single-cell suspension of 2 × 10^6^ transfected PC9 cells in 200 μL of sterilized PBS into the tail vein. After four weeks, we harvested the whole lung tissue of mice for further pathological analysis.

### RNA pull-down assay

Utilizing the TranscriptAid T7 High Yield Transcription Kit (Thermo, USA), RNA products were generated in vitro after the LINC00880 sequence was cloned into the pcDNA3.1 plasmid and biotinylated using the Pierce RNA 3’ End Desthiobiotinylation Kit (Thermo, USA). According to the Pierce Magnetic RNA-Protein Pull-down Kit (Thermo, USA) manufacturer’s instructions, 50 pmol of RNA was used for each sample to exert RNA pull-down assay.

### RNA immunoprecipitation (RIP) assay

According to Magna RNA-Binding Protein Immunoprecipitation Kit (Millipore, Bedford, MA) manufacturer’s instructions, RIP assay was carried out. The purified immunoprecipitated RNAs were then subjected to qRT-PCR following proteinase K digestion. Supplementary Table 3 list all the antibodies used for RIP.

### Co-immunoprecipitation (co-IP) assay

Centrifugation was performed to produce cell lysates at 13,000 rpm for 15 min at 4 °C. According to the manufacturer’s protocol, the Pierce Classic Magnetic IP/Co-IP Kit (Thermo, USA) with 4 primary antibodies (CDK1, PRDX1, FLAG, and MYC) were used for co-IP assay. Using the target antibodies, western blotting was used to examine the products. Supplementary Table 3 list all the antibodies used for co-IP.

### Chromatin immunoprecipitation (ChIP) assay

Lysates of PC9 cells were employed in the ChIP experiment. According to the manufacturer’s protocol, the Magna ChIP™ GChromatin Immunoprecipitation Kit (Merck Millipore, Germany) was used to carry out ChIP assay, using 5 primary antibodies (H3K27ac, FOXP3, STAT1, STAT4, and IgG). qRT-PCR was then used to analyze the ChIP pull-down DNA. Supplementary Tables 2, 3 list all the primers and antibodies used for ChIP.

### Dual-luciferase reporter assay

Dual-Luciferase Reporter Assay Kit (Vazyme, China) was used to perform the luciferase reporter assay in accordance with manufacturer’s protocol. Luminescence was evaluated on a GloMax plate reader (Promega, USA). Firefly and Renilla luciferase activities were measured, and the relative luciferase activity was normalized to Renilla luciferase activity.

### In vitro kinase activity assay

To assess endogenous kinase activity, anti-CDK1 antibodies was used to immunoprecipitate CDK1 from whole cell lysates. In kinase buffer, which has the following ingredients: 40 mM Tris (pH 7.5), 20 mM MgCl2, 0.1 mg/ml BSA, 2 mM DTT, 100 M Na3VO4, and 10 M ATP, the kinase assay was carried out. The kinase reactions were conducted using the acquired immunoprecipitates. The kinase substrate was histone H1 (1 mg per reaction). ADP-Glo™ Kinase Assay Kit (Promega, USA) was used to examine the kinase activity of CDK1 in accordance with manufacturer’s protocol.

### Immunohistochemistry (IHC) and RNA in situ hybridization (RNA-ISH)

According to the manufacturer’s protocol, staining and analysis for the IHC and RNA-ISH assay were carried out. The antibodies anti-FOXP3 (1:200, Proteintech), anti-p-CDK1(T161) (1:200, CST), anti-p-PRDX1 (1:200, CST) and anti-p-AKT (1:200, CST) were used. The probe for RNA-ISH labeled with digoxin (DIG) was synthesized by Pinuofei Biotechnology (Wuhan, China). The staining results were evaluated by the extent and intensity of all markers. Supplementary Table 3 list all the antibodies used for IHC.

### Bioinformatic analysis

RNA expression data and related clinical characteristics of TCGA and GTEx were downloaded using UCSC Xena. GSE29013, GSE37745 and GSE42127 (LUAD microarray dataset) was acquired from Gene Expression Omnibus (GEO). The ChIP-seq data of H3K27ac and H3K4me1 for A549 cells, PC9 cells, and NAL (normal adult lung) tissue were obtained as same as our previous study [7]. MACS2 was used for peak calling (parameters: -keepdup all -g hs -q 0.01). ROSE was used to carry out the calling of the SE. The default settings for every parameter were used. SEs having a length of < 2 kb detected by ROSE were removed. The GREAT online tool was utilized to discover genes associated with SEs according to the criterion: basal + extension: 1 kb upstream, 1 kb downstream, and 1000 kb max extension. IGV (version 2.9.4) and Juicebox (version 1.11.08) were used to create visualizations of these data.

### Statistical analysis

R (version 4.1.3) and GraphPad Prism (version 8.0.1) were used for performing statistical analysis. Survival curves was planted utilizing the Kaplan-Meier method, while the diversity was estimated by log-rank test. All experiments were carried out using ≥ 3 biological replicates. The results are exhibited as the mean ± S.D. Comparison between paired groups was evaluated by paired t-test, while in the unpaired groups, unpaired t-test was exerted. All statistical tests were two-sided. *P < 0.05, **P < 0.01, or ***P < 0.001 represent the statistical significance.

## Results

### The characteristics of LINC00880 in LUAD

**Fig. 1:**
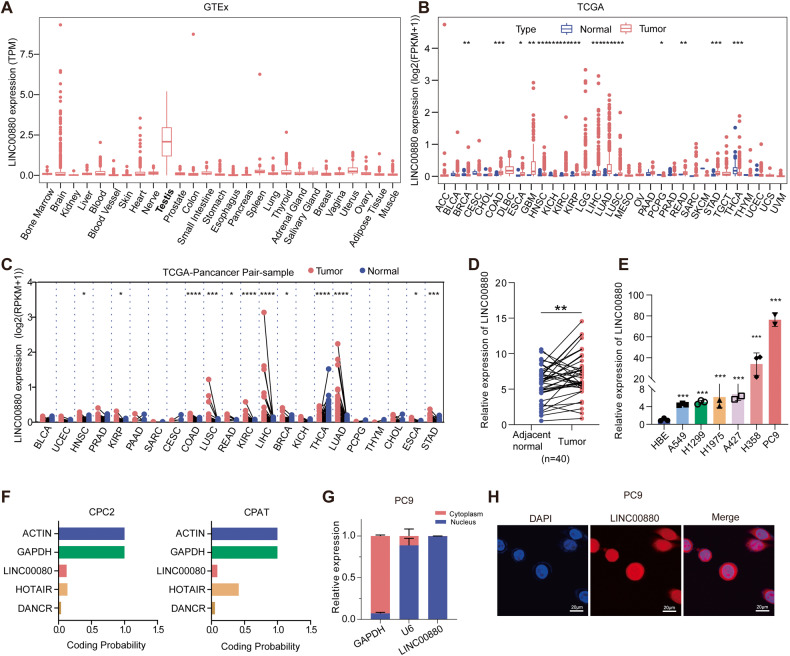
The characteristics of LINC000880 in LUAD. **A** Tissue-specific gene expression from GTEx data for LINC00880. **B** Pan-cancer gene expression of LINC00880 in tumor-normal unpaired samples from TCGA. **C** Pan-cancer gene expression of LINC00880 in tumor-normal paired samples from TCGA. **D** qRT-PCR detection of LINC00880 expression in LUAD (*n* = 40) tumor-normal paired tissues. **E** qRT-PCR detection of LINC00880 expression in multiple LUAD cells and in normal lung bronchial epithelial cell. **F** Coding potential was determined by Coding potential calculator 2 (CPC2) and Coding Potential Assessment Tool (CPAT). **G** qRT-PCR detection of LINC00880 expression in the cytoplasmic and nuclear fractions. **H** Subcellular localization of LINC00880 detected by FISH. Scale bar: 20 μm. The data are shown as the mean ± S.D. of at least three replicates (*P < 0.05, **P < 0.01, ***P < 0.001).

We previously characterized SE-associated lncRNAs (SE-lncRNAs) in LUAD specimens using a SE-lncRNAs microarray [7], among which, LINC00880 attracted our attention due to its markedly upregulated expression in tumor samples (Fig. S1A, B). GTEx database analysis showed that among normal human tissues, the expression of LINC00880 was the highest in the testis (Fig. 1A), and previous research had demonstrated that the sequence encoding LINC00880 was located at a genome-susceptible region (3q25.31) in testicular cancer [14]. The TCGA pan-cancer analysis revealed upregulated LINC00880 expression across most types of cancers, including head and neck squamous cell carcinoma (HNSC), liver hepatocellular carcinoma (LIHC), kidney renal clear cell carcinoma (KIRC), glioblastoma (GBM), and LUAD (Fig. 1B). Pan-cancer analysis of TCGA paired sample exhibited that LINC00880 was highly and widely expressed in most types of tumors, including LUAD (Fig. 1C). The ImmLnc dataset [15] also revealed that LUAD tumor tissues exhibited the most elevated expression of LINC00880 (Fig. S1C). Results of the qRT-PCR also validated that the LINC00880 was strongly expressed in tumor than the matched normal tissue (Fig. 1D), as well as in LUAD tumor cells when compared to the normal lung cell (Fig. 1E). RACE assay was utilized to uncover a 2,028 nt sequence for LINC00880, which was consistent with the existent transcript of LINC00880 (NR_034007.1) in the NCBI dataset, except for a 12 nt poly-A tail (Fig. S1E). Results of the oligo(dT)18 assay also suggest that LINC00880 contains a poly-A tail (Fig. S1F). The coding potential calculator 2 (CPC2) [16] and coding potential assessment tool (CPAT) [17], with two famous lncRNAs HOTAIR [18, 19] and DANCR [20] as positive control, predicted weak protein-coding potential for LINC00880 (Fig. 1F), which was further confirmed by phyloCSF [21] (Fig. S1D), and was consistent with the characteristics of lncRNAs. Moreover, results obtained by nuclear and cytoplasmic extraction and RNA FISH unveiled that LINC00880 was primarily distributed in the nucleus (Fig. 1G, H). Summarily, these findings imply that the LINC00880 is notably upregulated in LUAD.

### LINC00880 is a SE-driven lncRNA that is hijacked by FOXP3

**Fig. 2:**
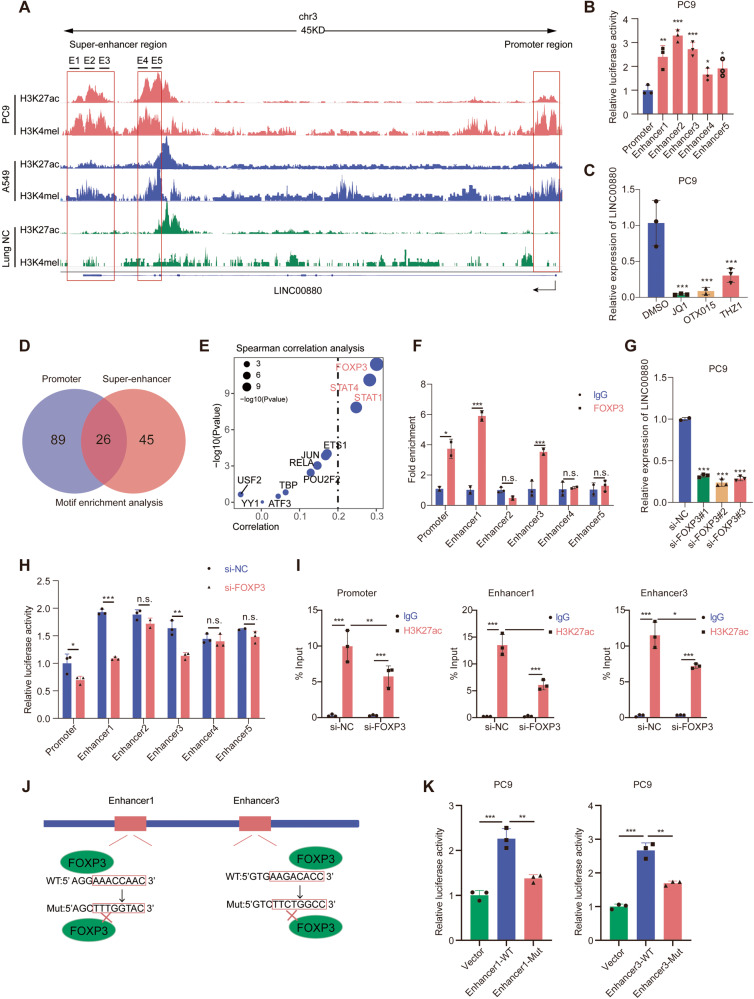
LINC00880 is a super-enhancer driven lncRNA hijacked by FOXP3. **A** Gene tracks depicting the gene body and super-enhancer region of LINC00880 in LUAD cells PC9 and A549, as well as normal lung tissue with measured H3K27ac and H3K4me1 marks. The data were retrieved from Encode project. **B** The luciferase activities of promoter and five enhancer elements were measured through dual-luciferase reporter assay in PC9 cells. **C** qRT-PCR detection of LINC00880 expression treated with JQ1 (0.5 μM), QTX015 (0.5 μM), and THZ1 (0.5 μM) for 1 h (left panel). Quantitation analysis (right panel). **D** Venn diagrams showing commonly binding transcription factors on the promoter and super-enhancer regions of LINC00880. **E** Spearman correlations analysis of LINC00880 expression with 10 commonly binding transcription factors on the promoter and super-enhancer regions of LINC00880. The data were acquired from TCGA-LUAD database. **F** ChIP analysis using antibodies against FOXP3, Immunoglobulin G (IgG) was used as the negative control (left panel) Quantitation analysis (right panel). **G** Detection of LINC00880 expression in PC9 cells treated with FOXP3 knockdown by qRT-PCR. **H** The luciferase activities of promoter and five enhancer elements of LINC00880 were measured in PC9 cells treated with FOXP3 knockdown. **I** The PC9 cells with FOXP3 knockdown were subjected to ChIP analysis using antibodies against H3K27ac. The association with the E1, E3 and promoter region of LINC00880 was quantified by qRT-PCR. **J** Schematic representation of the enhancer1 and enhancer3 of LINC00880 containing the wild-type FOXP3 motif binding sequence or the mutant alleles. **K** The luciferase activities of the wild-type or mutant-type enhancer1 and enhancer3 of LINC00880 were measured in PC9 cells. The data are shown as the mean ± S.D. of at least three replicates (*P < 0.05, **P < 0.01, ***P < 0.001, ns. no significance).

To dissect the relationship between LINC00880 transcription and its SE, we analyzed the H3K27ac and H3K4me1 ChIP-seq profiles of A549, PC9, and NAL (normal adult lung) tissue which identified an aberrant activated ~8 kb SE region across the gene body of LINC00880 (Fig. 2A). The activity of the SE was higher in tumor cells than normal, especially in PC9 cells, with more significant signals of both H3K27ac and active markers of the SE. To measure the activity of the identified SE region, we divided it into five regions (E1-E5) and cloned each constituent enhancer into enhancer-reporter vectors, which revealed that all five regions were active in the PC9 cell line (Fig. 2B). Moreover, the cells that were individually treated with the small-molecule epigenetic inhibitors THZ1, OTX015, and JQ1, (targeting BRD4, and CDK7, respectively, which comprise the element of the SE-complex), exhibited significantly depressed expression of LINC00880 (Fig. 2C). This reveals that LINC00880 is a SE-associated lncRNA.

It has been reported that SEs recruit transcription factors (TFs) to mediate interactions with the promoters and orchestrate transcriptional dysregulation in cancer cells [22]. Through motif enrichment analysis, we obtained 26 candidate TFs that simultaneously occupied the promoter and SE regions of LINC00880 (Fig. 2D). Moreover, correlation analysis was performed using the TCGA-LUAD dataset, revealing that the expression of FOXP3, STAT1, and STAT4 were positively related to LINC00880 expression (spearman’s correlation of > 0.2) (Fig. 2E, Fig. S2A). Additionally, ChIP-qPCR was performed on FOXP3, STAT1, and STAT4 to assess their occupancy in both the promoter and SE regions of LINC00880, and the results revealed a significant enrichment of FOXP3 both in the promotor region and the E1 and E3 regions of the SE, but no enrichment was found for STAT1 or STAT4 (Fig. 2F, Fig. S2B). Inhibiting FOXP3 expression effectively suppressed the expression of LINC00880 (Fig. 2G, Fig. S2C) and weakened the activity of the promoter, E1, and E3 regions, as determined by the dual-luciferase reporter assay (Fig. 2H). We also decreased the expression of STAT1 and STAT4 by siRNA, respectively, and detected the mRNA expression of STAT1, STAT4, as well as LINC00880. And we found that LINC00880 expression was significantly attenuated by absence of STAT1 but not STAT4. (Fig. S2D–G). We then profiled H3K27ac signals in the promoter, E1, and E3 regions, which demonstrated that the absence of FOXP3 significantly decreased the acetylation modification of both the SE and promoter on H3K27 sites (Fig. 2I). Additionally, mutated E1 and E3 luciferase reporter gene plasmids were designed (Fig. 2J). Cells transfected with these mutated reporters showed decreased activity when compared to the wild-type control (Fig. 2K) [23]. Together, these findings demonstrate that the upregulation of LINC00880 is driven by its SE hijacking the transcription factor FOXP3, especially in the SE regions E1 and E3.

### LINC00880 sustains the malignant progression of LUAD both in vitro and in vivo

**Fig. 3:**
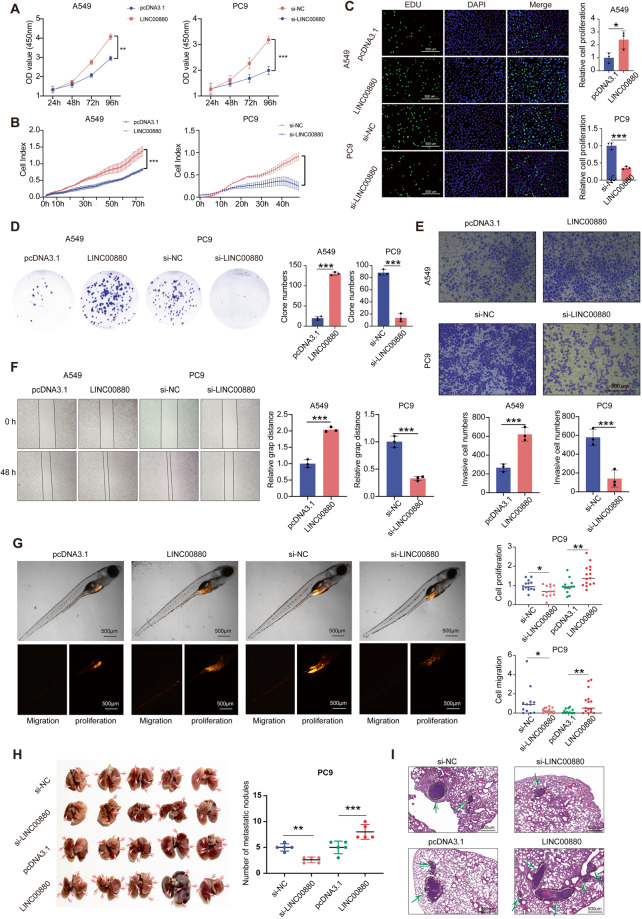
LINC00880 sustains malignant progression of LUAD in vitro and in vivo. **A**, **B** CCK-8 and real-time cell analysis (RTCA) assays showing LINC00880 overexpression promoted cell proliferation and LINC00880 knockdown significantly decreased cell proliferation. **C** Number of proliferating LINC00880 overexpression and knockdown cells, as determined by the EdU incorporation assay. DAPI was used to stain the nuclei. Scale bars: 500 μm (left panel). Quantitation analysis (right panel). **D** Colony formation of A549 cells with LINC00880 overexpression or PC9 cells with LINC00880 knockdown (left panel). Quantitation analysis (right panel). **E** The invasion of LINC00880 overexpression A549 cells assessed by transwell Scale bars: 500 μm (up panel). Quantitation analysis (down panel). **F** The migration of LINC00880 knockdown PC9 cells assessed by wound healing (left panel). Quantitation analysis (right panel). **G** Representative images of tumor detection and metastatic loci in the zebrafish tumor model (left panel). Quantitation analysis (right panel); (Cell proliferation: Si-NC *n* = 14; si-LINC00880 *n* = 13; pcDNA3.1 *n* = 13; LINC00880 *n* = 15; Cell migration: Si-NC *n* = 12; si-LINC00880 *n* = 14; pcDNA3.1 *n* = 13; LINC00880 *n* = 15). **H** Images of metastatic loci in the lung derived from mouse model; (left panel). Quantitation analysis (right panel); (Si-NC *n* = 5; si-LINC00880 *n* = 5; pcDNA3.1 *n* = 5; LINC00880 *n* = 5). **I** H&E stained detection of sections from lung metastasis nude mouse model. Scale bars: 500 μm. The data are shown as the mean ± S.D. of at least three replicates (*P < 0.05, **P < 0.01, ***P < 0.001).

To investigate the biological functions of LINC00880 in LUAD cells, assay both in vitro and in vivo were carried out. PC9 cells applies pooled siRNAs, because of its high expression level of LINC00880, while LINC00880 overexpression was used in A549 because of its low expression level of LINC00880, and the efficiencies of both models were confirmed (Fig. S3A, B). We found that the knockdown of LINC00880 significantly decreased cell proliferation, while the overexpression of LINC00880 promoted cell proliferation, which is determined by the results of the CCK8, RTCA, and EdU assays (Fig. 3A–C). Colony formation was also performed to reveal that LINC00880 was positively correlated with clonogenicity (Fig. 3D). Moreover, Transwell, wound scratch healing, and Matrigel invasion assays were used to estimate LUAD cell migration and invasion capacities, and the results showed that the lack of LINC00880 drastically inhibited cell migration and invasion. (Fig. 3E, F). To further validate these observations in vivo, the zebrafish tumor model was utilized to analyze tumor development and metastasis, specifically to understand the metastatic process mediated by LINC00880. In agreement with the in vitro results, the lack of LINC00880 dramatically weakened the proliferation and metastasis capacity (Fig. 3G). Additionally, we also constructed lung metastasis model, and the analysis of lung metastatic colonies showed that LINC00880 drastically promoted tumor metastasis (Fig. 3H, I). These data demonstrate that LINC00880 acts as an oncogenic lncRNA to sustain the malignant progression of LUAD in vitro and in vivo.

### LINC00880 interacts with CDK1 to enhance its kinase activity

**Fig. 4:**
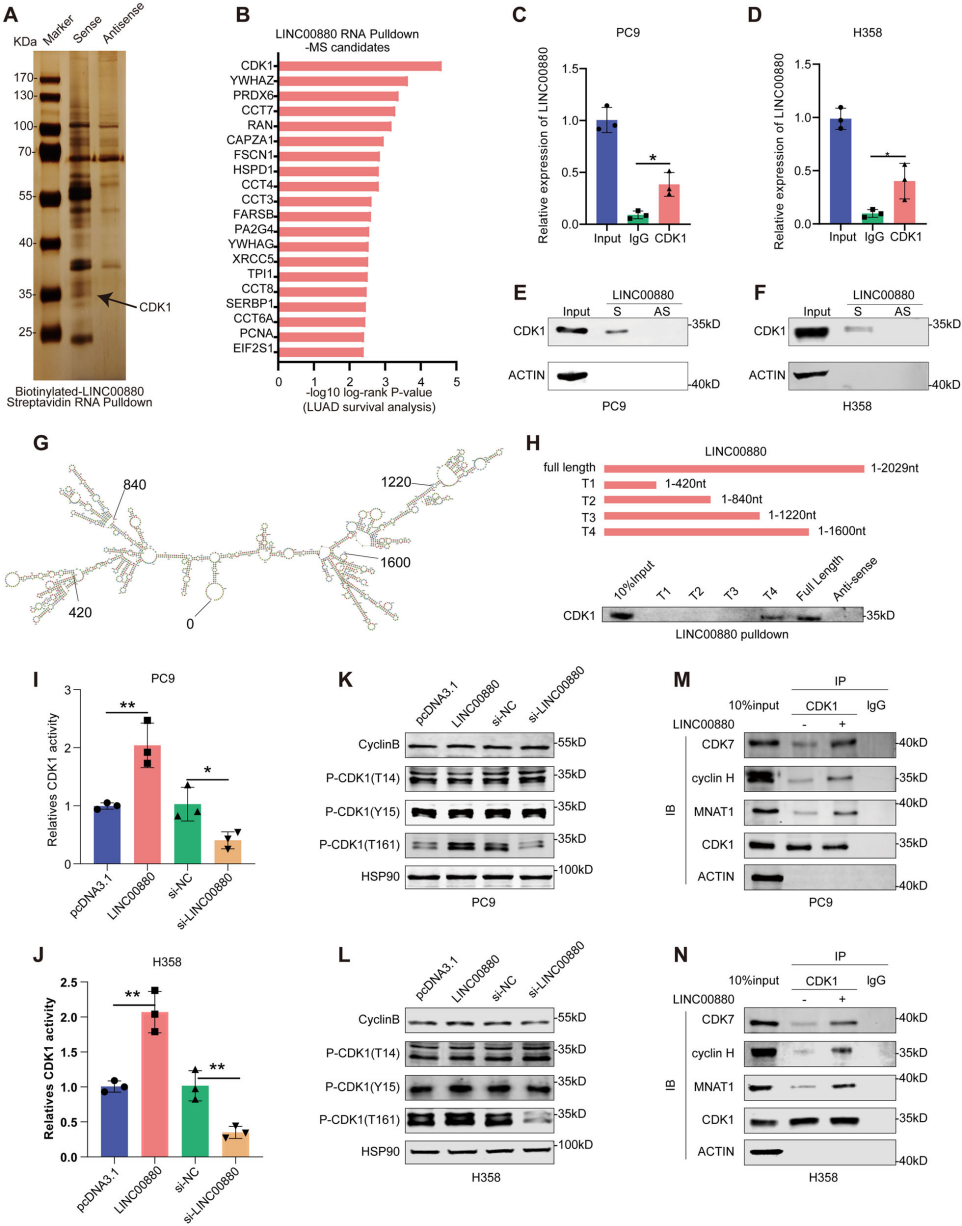
LINC00880 interacts with CDK1 to enhance its kinase activity. **A** Identification of LINC00880-interacting proteins. LINC00880 and the antisense of LINC00880 were synthesized and biotinylated in vitro followed by the RNA pull-down assay. The silver staining reveals the specific bands that were subjected to MS analysis (indicated with arrow). **B** LINC00880 RNA pulldown-MS candidates sorted for log-rank *P*-value in TCGA-LUAD patient survival analysis. **C**, **D** The interaction between LINC00880 and CDK1 was determined by a RIP assay using a CDK1-specific antibody, immunoglobulin G (IgG) was used as the negative control. **C**: PC9, **D**: H358. **E**, **F** By the RNA pull-down assay, CDK1 is pulled down with LINC00880, but not with the antisense of LINC00880. **E**: PC9, **F**: H358. **G** LINC00880 is predicted to have four stem-loop structures by using Rfold. **H** Immunoblot detection of the CDK1 protein in PC9 cells as retrieved by in vitro transcribed biotinylated RNAs of different constructs of LINC00880 or its antisense sequence (as negative control). **I**, **J** Activity of CDK1 in cell lysates was measured using a Histone H1 kinase substrate. **I**: PC9, **J**: H358. **K**, **L** Western blotting detection of cyclin B and phosphorylated CDK1 (T14/Y15/T161) expression in PC9 cells transfected with pcDNA3.1, LINC01977, si-NC, si-LINC00880. **K**: PC9, **L**: H358. **M**, **N** Co-immunoprecipitation (co-IP) of CDK1 with CAKs in PC9 cells transfected with LINC00880. **M**: PC9, **N**: H358. The data are shown as the mean ± S.D. of at least three replicates (*P < 0.05, **P < 0.01, ***P < 0.001).

It’s well known that lncRNAs can bind to proteins to perform their biological functions [24, 25]. To identify the potential proteins that interact with LINC00880, we carried out an RNA pull-down of LINC00880 and used mass spectrometry to analyze its precipitates. Following silver staining results suggest the proteins located at 35 kDa region may interact with LINC00880. (Fig. 4A). Based on the cover percentages and unique peptides, we selected the top 50 ranked proteins by mass and performed a survival analysis of TCGA-LUAD, among which, CDK1 attracted our attention for both its enrichment in the mass spectrometry analysis and negative correlation with LUAD prognosis (Fig. 4B, Table S4). The RIP and RNA pull-down assay results also validated the binding between LINC00880 and CDK1 in PC9 and H358 (Fig. 4C–F). To identify the LINC00880 nucleotide sequence that interacts to CDK1, according to stem-loop structures, we constructed truncations of LINC00880 (Fig. 4I). The results suggest that deleting the RNA fragment ranging from 1220 to 1600 dramatically reduced the binding ability of LINC00880 to CDK1, revealing that this region is critical for their interaction (Fig. 4H).

To clarify the impact of LINC00880 on CDK1, we performed the qRT-PCR and western blot assays, which indicates that neither the overexpression nor the knockdown of LINC00880 significantly affected the expression of CDK1 in terms of either mRNA or protein levels (Fig. S4A, B). CDK1, known as a member of the cyclin-dependent kinase family, is largely dependent on kinase activity to perform its functions [26]. The ADP-Glo Kinase Assay Kit was used to detect the kinase activity of CDK1 [27], which uncovered that the overexpression of LINC00880 significantly elevated the kinase activity of CDK1, while knocking down LINC00880 had the opposite effect (Fig. 4I, J). The kinase activity of CDK1 was determined by the phosphorylation state of its antibody pT161, as well as the dephosphorylation status of its antibodies pT14 and pY15 [28–30]. Cyclin B can interact with CDK1 to form a complex to control mitosis, and a higher expression of cyclin B can promote the activity of CDK1 [31]. Interestingly, perturbing LINC00880 expression had a significant impact on only pT161-CDK1 expression, but not on pT14-CDK1, pY15-CDK1, or cyclin B expression (Fig. 4K, L). The T161 site, which located at the T-loop of CDK1, is phosphorylated by CDK-activating kinases (CAKs), including CDK7, MNAT1, and cyclin H [32]. The co-IP assay found that the overexpression of LINC00880 facilitated interactions between CDK1 and CAKs (Fig. 4M, N), while the knockdown or overexpression of LINC00880 had little effect on the expression of the main components of CAKs (Fig. S4C). These observations suggest that LINC00880 promotes CDK1 kinase activity by increasing the phosphorylation level of pT161-CDK1 [29].

We further investigated whether the oncogenic properties of LINC00880 depend on the kinase activity of CDK1. We employed the overexpression of CDK1, the use of siRNAs targeting CDK1, and the specific small molecule inhibitor of pT161-CDK1 (RO-3306) (Fig. S4D–F). The results suggested that the overexpression of CDK1 partially rescued the reduce in the malignant phenotype observed in cells subjected to LINC00880 knockdown, while the oncogenic function of LINC00880 was abolished with either the knockdown of CDK1 expression or the inhibition of CDK1 kinase activity (Fig. S4G, H). Collectively, these results indicate that the contribution of LINC00880 to malignant progression depends on CDK1 kinase activity.

### LINC00880 regulates the PTEN-AKT signaling pathway via CDK1-dependent p-PRDX1 inactivation

**Fig. 5:**
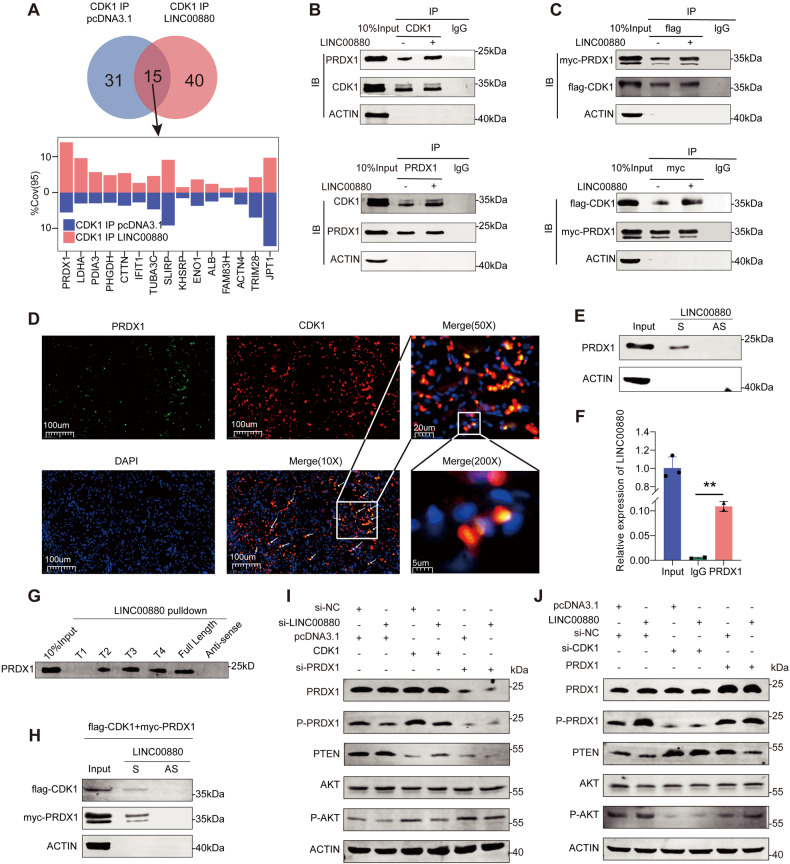
LINC00880 regulated PTEN-AKT pathway though forming a complex with CDK1 and PRDX1. **A** Venn diagram showing the number of protein binding to CDK1 after LINC00880 overexpression (up panel). Changes of %Cov(95) (down panel). **B** Co-IP assays showed that endogenous CDK1 interacted with endogenous PRDX1, and LINC00880 enhanced the interaction between CDK1 and PRDX1. **C** PC9 cells were transfected with combinations of plasmids encoding Myc-PRDX1 and Flag-CDK1 followed by co-IP assays. **D** represent Immunofluorescence images showing the Co-localization of CDK1 and PRDX1 in LUAD tissues. **E** RNA pull-down and western blot assays were used to evaluate the interaction between LINC00880 and PRDX1. **F** RIP and qRT-PCR assays were used to measure the enrichment of LINC00880. **G** Immunoblot detection of the PRDX1 protein in PC9 cells as retrieved by in vitro transcribed biotinylated RNAs of different constructs of LINC00880 or its antisense sequence (as negative control). **H** RNA pull-down and western blot assays were used to evaluate the interaction between LINC00880 and flag-CDK1 and myc-PRDX1. **I**, **J** Western blotting detection of PRDX1, p-PRDX1, PTEN, AKT, p-AKT levels in PC9 cells transfected. The data are shown as the mean ± S.D. of at least three replicates (*P < 0.05, **P < 0.01, ***P < 0.001).

CDK1 exerts its biological effect by phosphorylating different substrates [13, 33–37]. To determine the downstream target of LINC00880, we performed a CDK1 co-IP with LC/MS in LINC00880-overexpressed LUAD cells. From the results, we found that overexpressing LINC00880 promoted the interaction of CDK1 with PRDX1, which was selected as the candidate downstream target of LINC00880 (Fig. 5A). The co-IP assay confirmed that the presence of LINC00880 facilitated the interaction between CDK1 and PRDX1 (Fig. 5B). Moreover, a tag-labeled fusion protein was utilized to demonstrate that increased LINC00880 expression promoted the protein binding between CDK1 and PRDX1 (Fig. 5C). The results of the co-localization analysis also supported these observations, revealing that CDK1 strongly co-localized with PRDX1 (Fig. 5D, Fig. S5A). Interestingly, the previous LINC00880 RNA pull-down results show that LINC00880 could also interact with PRDX1 (Fig. S5B). Then, we performed RNA pull-down and RIP assays to validate the binding between PRDX1 and LINC00880 (Fig. 5E, F). Subsequently, truncated LINC00880 RNA pull-down was performed to confirm the nucleotide sequence responsible for the binding of LINC00880 to PRDX1, and the results suggested that deleting RNA fragment ranging from 420 to 840 dramatically reduced the binding of LINC00880 to PRDX1 (Fig. 5G). This result also indicated that the positive chain of LINC00880, but not the antisense chain, enables it to bind to CDK1 and PRDX1 (Fig. 5H). The above results demonstrated that LINC00880 act as a scaffold to improve the binding between CDK1 and PRDX1, which form the RNA-protein ternary complex.

Further, we found that perturbing LINC00880 expression had little impact on the expression of PRDX1, while significantly influencing the phosphorylation level of PRDX1 (p-PRDX1) (Fig. 5I, J), which is the inactivated form of PRDX1 [38]. The knockdown and overexpression of LINC00880 significantly decreased and increased the expression level of p-PRDX1, respectively, as well as key molecules involved in the downstream pathways of PRDX1, including the PETN and AKT signaling pathways [39] (Fig. 5I, J). The elevation in p-PRDX1 induced by CDK1 overexpression was also attenuated when inhibiting LINC00880 expression (Fig. 5I). Meanwhile, the knockdown of CDK1 expression or the dampening of its activity abolished the influences of both the phosphorylation level of PRDX1 and the downstream signaling pathway of PRDX1 that was caused by LINC00880 (Fig. 5J, Fig. S5C). Additionally, decreasing the expression or inhibiting the activity of PTEN (SF1670) hampered the oncogenic properties of LINC00880 (Fig. S5D). Taken together, these results exhibited that LINC00880 function as a scaffold to facilitate the binding between CDK1 and PRDX1, which then affects the phosphorylation level and downstream signaling pathway of PRDX1.

### LINC00880 is a hopeful prognostic predictor in early-stage LUAD

**Fig. 6:**
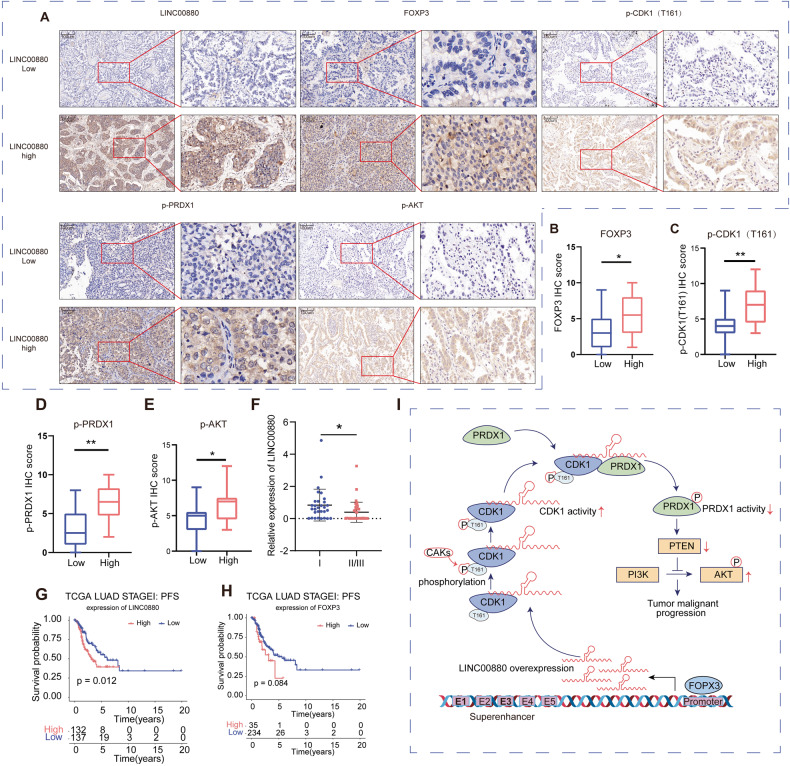
LINC00880 is a promising predictor of poor outcome in early-stage LUAD. **A** Representative image of RNA in situ hybridization (RNA-ISH) of LINC00880 and Immunohistochemistry (IHC) staining sections of patients with LUAD. FOXP3, p-CDK1, p-PRDX1 and p-AKT was significantly high expressed at high LINC00880 expression LUAD patients. Scale bars: 100 μm. **B**–**E** FOXP3, p-CDK1 (T161), p-PRDX1 and p-AKT IHC score between low and high LINC00880 expression LUAD patients. **F** Relative expression of LINC00880 detected by qRT-PCR from different pathological stage of LUAD in our cohort. **G**, **H** TCGA-LUAD PFS survival analysis in stage I patients according to the expression of LINC00880 and FOXP3. **I** Graphic abstract. Figure in I was created using Adobe Illustrator.

Next, we measured LINC00880 expression in LUAD tissues obtained from 152 patients, which were split into two groups based on LINC00880 expression level (Table S1). Subsequently, the expression of FOXP3, pT161-CDK1, p-PRDX1 and p-AKT were also detected in these two groups by IHC, and the results suggested that FOXP3, pT161-CDK1, p-PRDX1 and p-AKT were significantly elevated in the LINC00880-high group (Fig. 6A–E). Additionally, the relationship between the LINC00880 expression and the clinicopathological information was analyzed in this cohort. Interestingly, early-stage LUAD (Stage I) had a high LINC00880 expression, which was also confirmed in GSE29013 (Fig. 6F, S6A). The high expression of LINC00880 was also revealed to be strongly related to a poor prognosis in early-stage LUAD by the use of progression free survival (PFS) analysis in TCGA LUAD Stage I patients (Fig. 6G). However, as the clinical stage proceeded, the predictive power declined (Fig. S6B, C). We also found that higher expression levels of FOXP3, CDK1 and PRDX1 are associated with poorer prognosis (Fig. 6H, S6D–G). Collectively, we confirmed that LINC00880 functions to cause bad prognosis in the early stage LUAD.

## Discussion

Recently, many progressions have made in LUAD treatment, but which prognosis remains poor. Identifying novel target genes may give impetus to the development of novel LUAD therapies. Previous studies have reported that SE regulates the transcriptional processes to mediate the transcriptional dysregulation that is characteristic of tumorigenesis. However, little is known about the dysregulated lncRNAs that are mediated by activated SEs (SE-lncRNAs) in LUAD. In this study, we confirmed that a SE-lncRNA, known as LINC00880, is regulated by the transcription factor FOXP3 through its binding to the promoter and SE regions of LINC00880. More interestingly, the SE was found to share partial overlap with the genomic region of LINC00880. We also found that LINC00880 expression was higher in stage I LUAD patients when compared to stage II/III LUAD patients, and that high LINC00880 expression was related to poor outcomes in stage I LUAD patients, suggesting that LINC00880 was a promising prognostic marker for LUAD. Mechanistically, LINC00880 can act as a scaffold to improve the interaction between CDK1 and PRDX1, ultimately resulting in a marked increase in the phosphorylation of PRDX1 by activated CDK1. This phosphorylated form of PRDX1 consequently loses its activity, thereby dysregulating its downstream signaling pathways. Our results therefore provide an epigenetic perspective from which to explain the tumorigenesis of LUAD, especially in early stage (Fig. 6K).

SEs are large clusters of adjacent enhancers that are enriched with TFs, co-factors, and epigenetic modifications [40]. Recent studies have confirmed that SEs play an essential role in cancer. Mechanically, TFs are recruited by SEs, and they subsequently cause histone modifications on nearby nucleosomes, such as H3K27ac and H3lys4-methylation, to occur (H3K4me1) [41]. SE can regulate gene expression by forming core transcriptional regulatory circuitry (CRC) [6, 42]. The role of SE-lncRNAs in cancer attracted our attention. Our previous study found that SE-hijacked LINC01977 performed indispensable biological functions in TGF-β abundant microenvironment induced by TAM2 infiltration [7]. However, the role of SE-lncRNA in the context of LUAD has been unknown until now. Thus, in this study, we investigated the SE-lncRNA known as LINC00880, which contained a locus marked by a SE, and its treatment with SE inhibitors significantly repressed the expression of LINC00880. Results of the dual-luciferase reporter assay indicated that the elements of the SE enhance the expression of LINC00880 to varying degrees. These findings suggest that LINC00880 is driven by its SE. Much evidence supports that the functions of SEs depend on their cooperative interactions between TFs, and that transcriptional activation caused by SEs with greater numbers of TF-binding sites are more susceptible to changes in TF concentrations [4, 22, 43–45]. Furthermore, we observed that LINC00880 was transcriptionally driven by the transcription factor FOXP3. Using the TCGA database, the expression of LINC00880 was confirmed to be positively correlated with FOXP3, and we found that the knockdown of FOXP3 significantly reduced LINC00880 expression. Moreover, we discovered that FOXP3 directly bound to the promoter, E1, and E3 regions of LINC00880 and enhanced their H3K27ac levels, ultimately activating the transcription of LINC00880.

LncRNAs can exercise their biological functions by binding to proteins to regulate both their localization and stability, affect the assembly of protein complexes, and influence the separation of proteins from such complexes. For instance, lncRNA AGPG, through binding to PFKFB3 and then enhancing PFKFB3 stability, has a great influence in promoting glycolysis and cell proliferation, thereby facilitating the evolution of esophageal squamous cell carcinoma (ESCC) [46]. To understand the molecular mechanism involved in the promotion of LUAD progression by LINC00880, we performed an RNA pull-down assay followed by mass spectrometry. Consequently, we identified that LINC00880 interacted with CDK1 directly through the RNA pull-down assay and RIP assay. CDK1 not only acts as a cell cycle regulator, but also participates in transcriptional regulation, epigenetic regulation, DNA damage repair, proteolytic degradation, metabolism, and stem cell self-renewal [12]. TCGA database showed that high CDK1 expression correlates with poor overall survival in LUAD. In this study, we demonstrated that LINC00880 had no significant effect on the mRNA or protein expression of CDK1, but we found that it did enhance the kinase activity of CDK1. It has been generally acknowledged that the kinase activity of CDK1 mainly depends on the binding of cyclin B, the phosphorylation of T161 in CDK1, and the dephosphorylation of T14 and Y15 in CDK1 [30, 47–49]. Through further research, we found that LINC00880 enhanced the kinase activity of CDK1 by promoting CAKs phosphorylating the activating phosphorylation sites (threonine 161) in CDK1, which is responsible for improving both substrate binding and complex stability.

CDK1 has been shown to facilitate tumorigenesis by interacting and phosphorylating different substrate proteins, such as SOX2, hTERT, and HUR, which is beyond cell cycle control [13, 50, 51]. Subsequently, we determined that CDK1 bound to PRDX1 and that LINC00880 overexpression enhanced their interaction. Then, we demonstrated that there exist interaction events between LINC00880 and PRDX1. It has been reported that lncRNAs are able to mediate the assembly of protein complexes in a scaffolding manner. For example, both ends of the lncRNA known as HOTAIR have been shown to bind to PRC2 complex and LSD1, respectively, and then assemble these two different histone modifiers into a protein complex. Likewise, in the current study, we found that CDK1 and PRDX1 bound to the different fragments of LINC00880, respectively, and that LINC00880 allowed for robust interaction between CDK1 and PRDX1. CDK1 then phosphorylated PRDX1, thereby causing the loss of PRDX1 activity, and ultimately regulating the PTEN/AKT pathway. Moreover, we showed that CDK1 knockdown or treatment with a CDK1 inhibitor (RO3306) decreased cell proliferation, while CDK1 overexpression partially rescued the growth-inhibitory effect of LINC00880 knockdown. Collectively, these findings delineate the mechanism of LINC00880-promoted LUAD progression as one that involves the forming of a complex between itself, CDK1, and PRDX1 in a scaffolding manner.

Our study reveals that LINC00880, which is transcriptionally driven by the transcription factor FOXP3 via the LINC00880 SE, promotes cell growth, invasion, and metastasis by forming a complex with CDK1 and PRDX1, and thus regulating the PTEN-AKT pathway, ultimately facilitating the malignancy of LUAD. We also demonstrated that the SE-lncRNA LINC00880 is dependent on pT161-CDK1 for promoting tumor progression, which provides a new perspective from which to achieve the specific activation of CDK1 without affecting its expression. In conclusion, our findings firstly identify LINC00880 as a candidate oncogene in LUAD, and LINC00880 may serve as a promising clinical diagnostic biomarker for early-stage LUAD.

## Availability of data and material

All data generated or analyzed in this study are included in the article and its additional files. TCGA and GTEx dataset can be acquired from the UCSC Xena (https://xenabrowser.net/). GEO dataset was acquired from Gene Expression Omnibus (ncbi.nlm.nih.gov/geo/). ENCODE dataset was acquired from the website (http://encodeproject.org/), and ImmLnc dataset were was acquired from the website (http://bio-bigdata.hrbmu.edu.cn/ImmLnc/). LncRNA microarray data have been submitted to the GEO database (accession number GSE196584) in our previous study [7]. The processed data are available from the corresponding author upon reasonable request.

### Reporting summary

Further information on research design is available in the Nature Research Reporting Summary linked to this article.

## Supplementary information

Figure. S1

Figure. S2

Figure. S3

Figure. S4

Figure. S5

Figure. S6

Reporting Summary

Supplementary-Table

western_blot_supplement

Figure legends
