# Supplementary figures and images for: The super-enhancer-driven lncRNA LINC00880 acts as a scaffold between CDK1 and PRDX1 to sustain the malignance of lung adenocarcinoma
Source: Cell Death Dis. 2023 Aug 24;14(8):551. doi: 10.1038/s41419-023-06047-w (PMC10449921; doi:10.1038/s41419-023-06047-w)

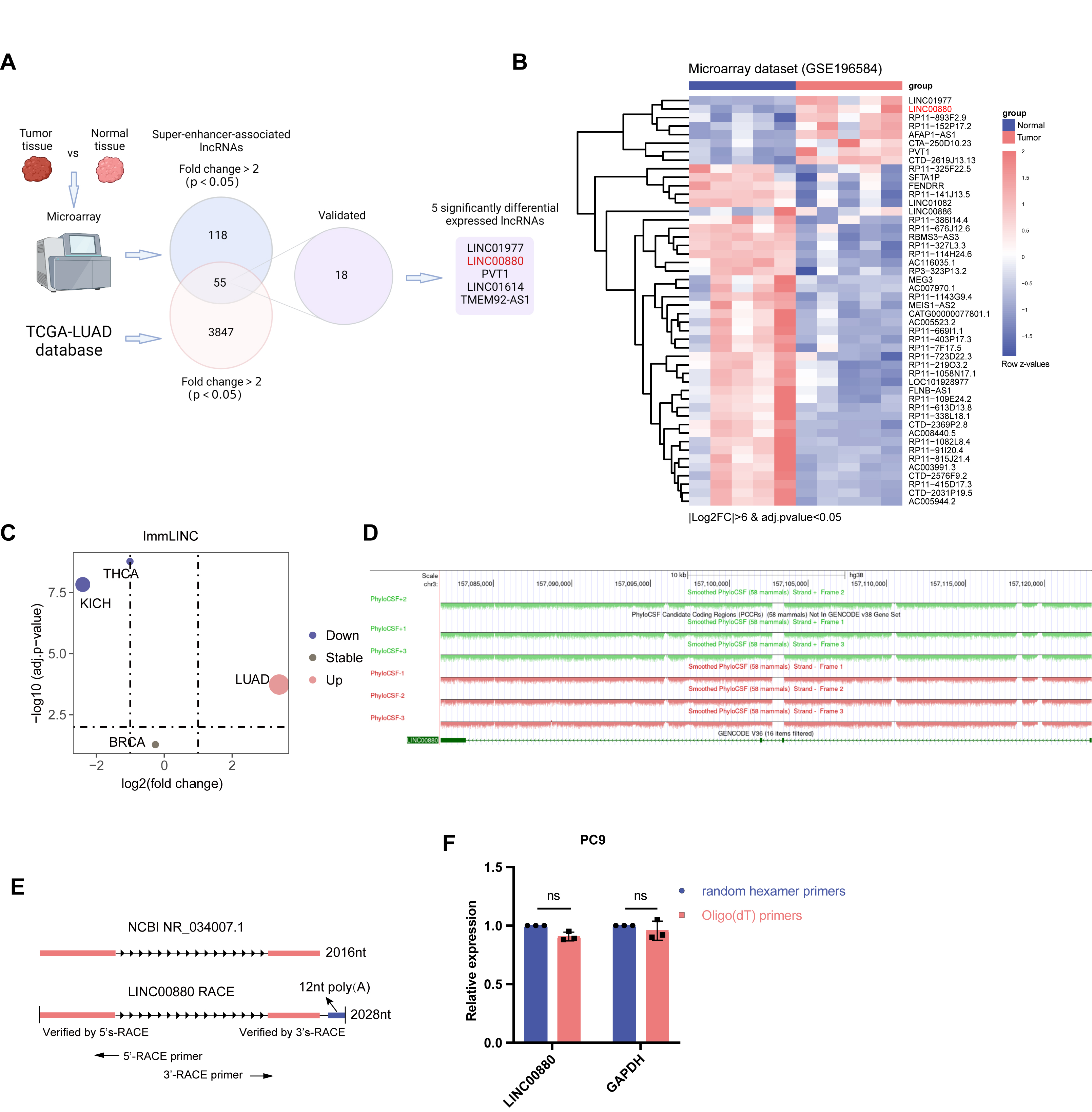

Supplement: Supplementary file 1 — Figure. S1 [file 41419_2023_6047_MOESM1_ESM.jpg]

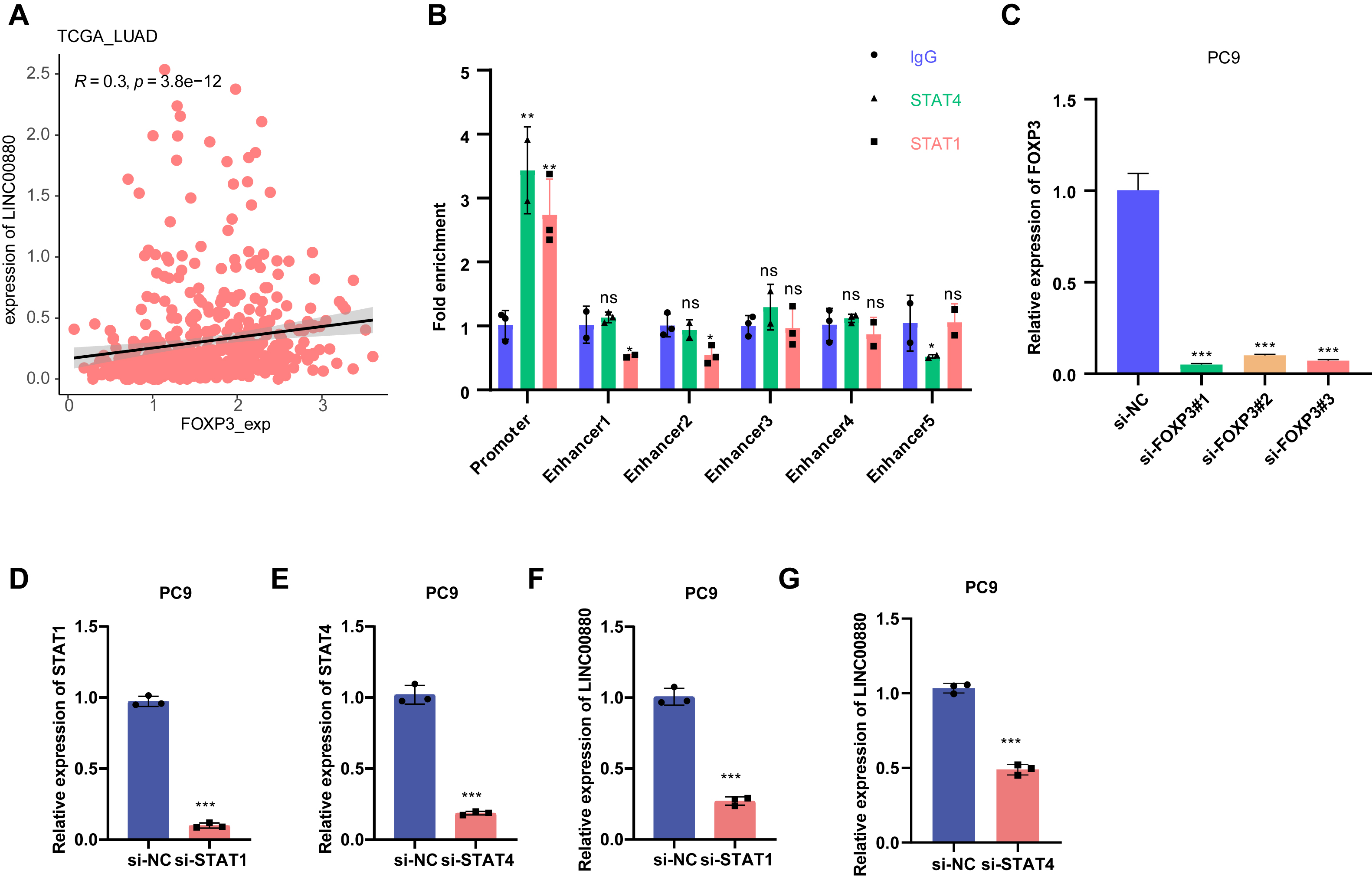

Supplement: Supplementary file 2 — Figure. S2 [file 41419_2023_6047_MOESM2_ESM.jpg]

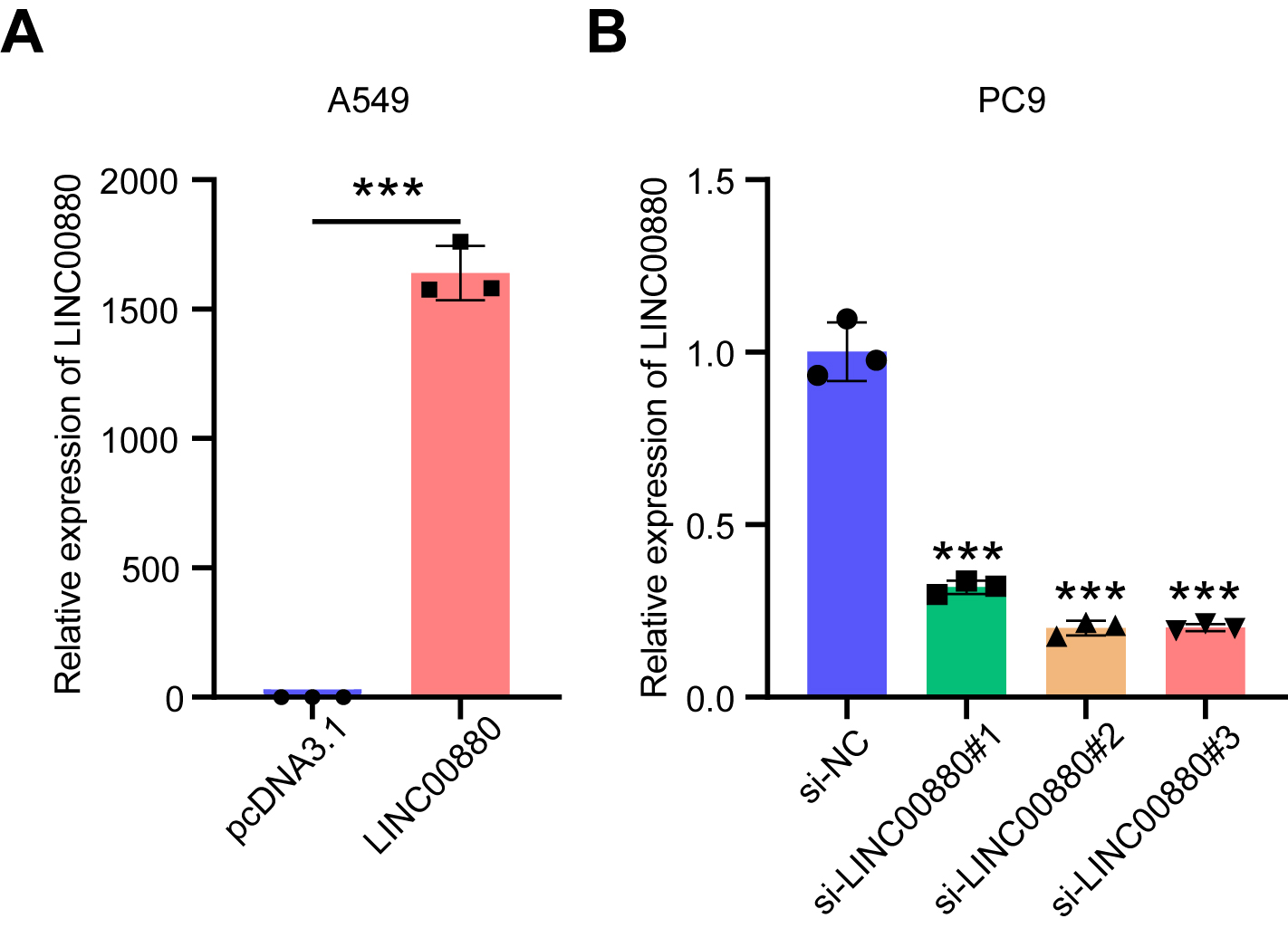

Supplement: Supplementary file 3 — Figure. S3 [file 41419_2023_6047_MOESM3_ESM.jpg]

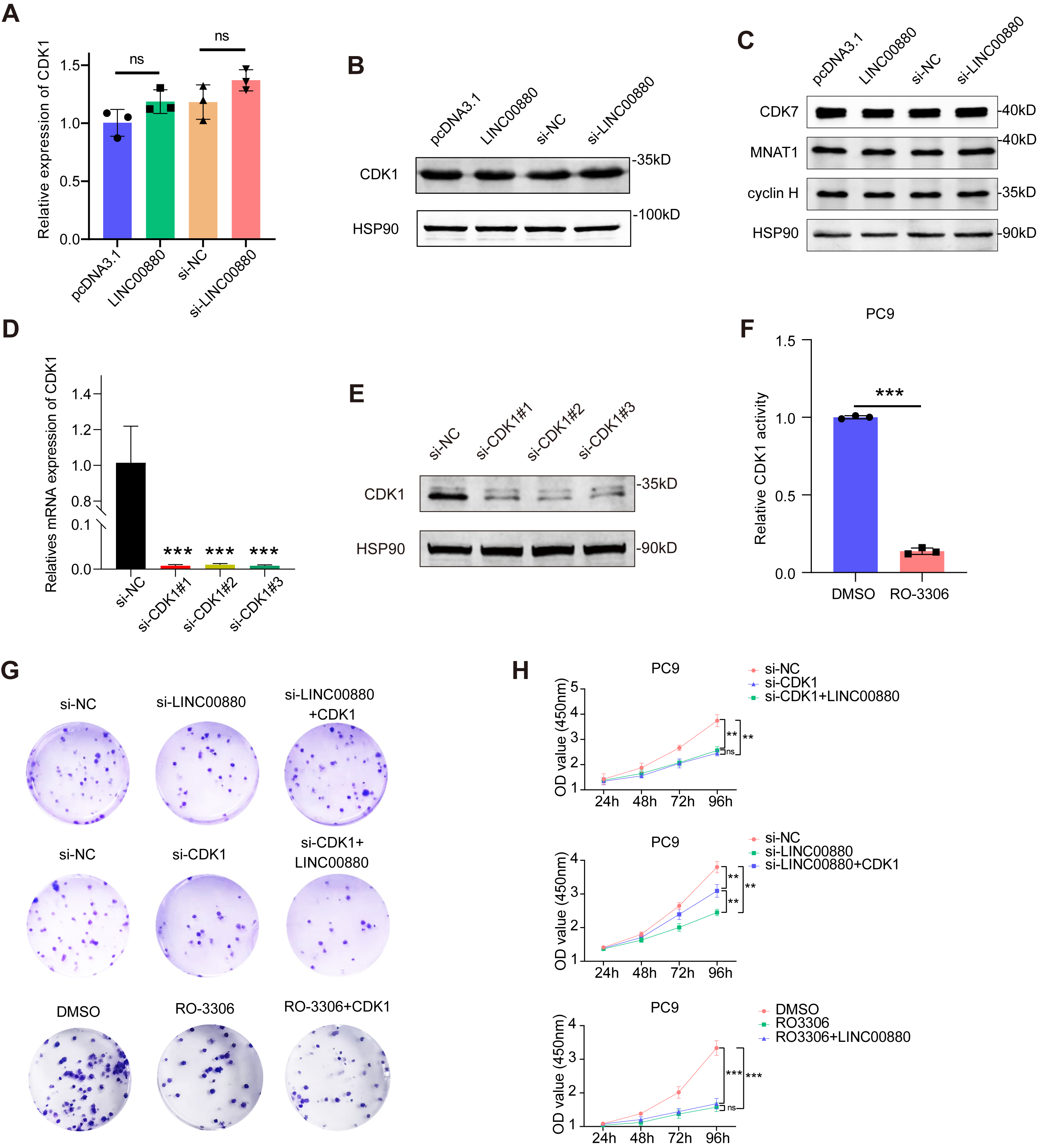

Supplement: Supplementary file 4 — Figure. S4 [file 41419_2023_6047_MOESM4_ESM.jpg]

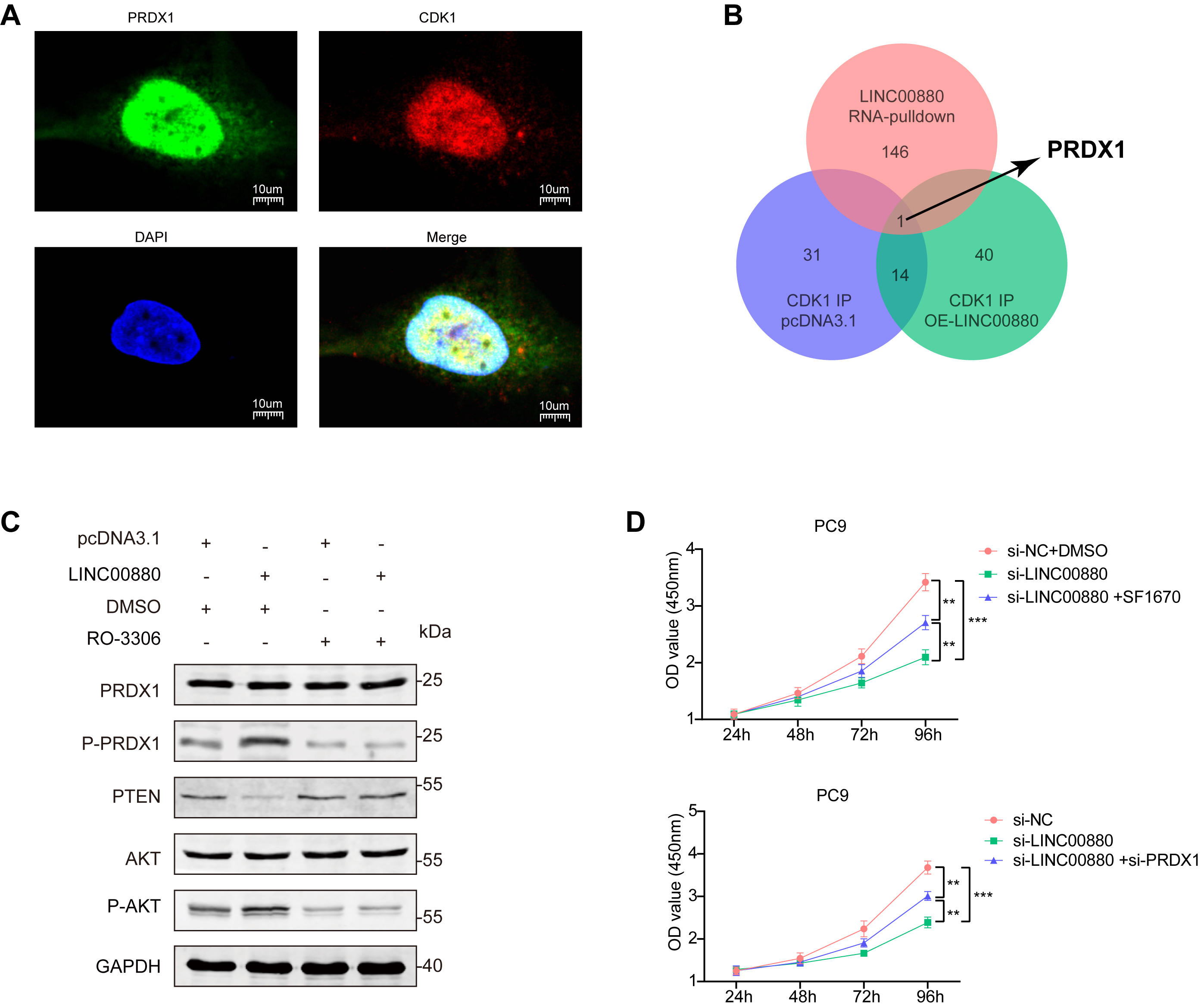

Supplement: Supplementary file 5 — Figure. S5 [file 41419_2023_6047_MOESM5_ESM.jpg]

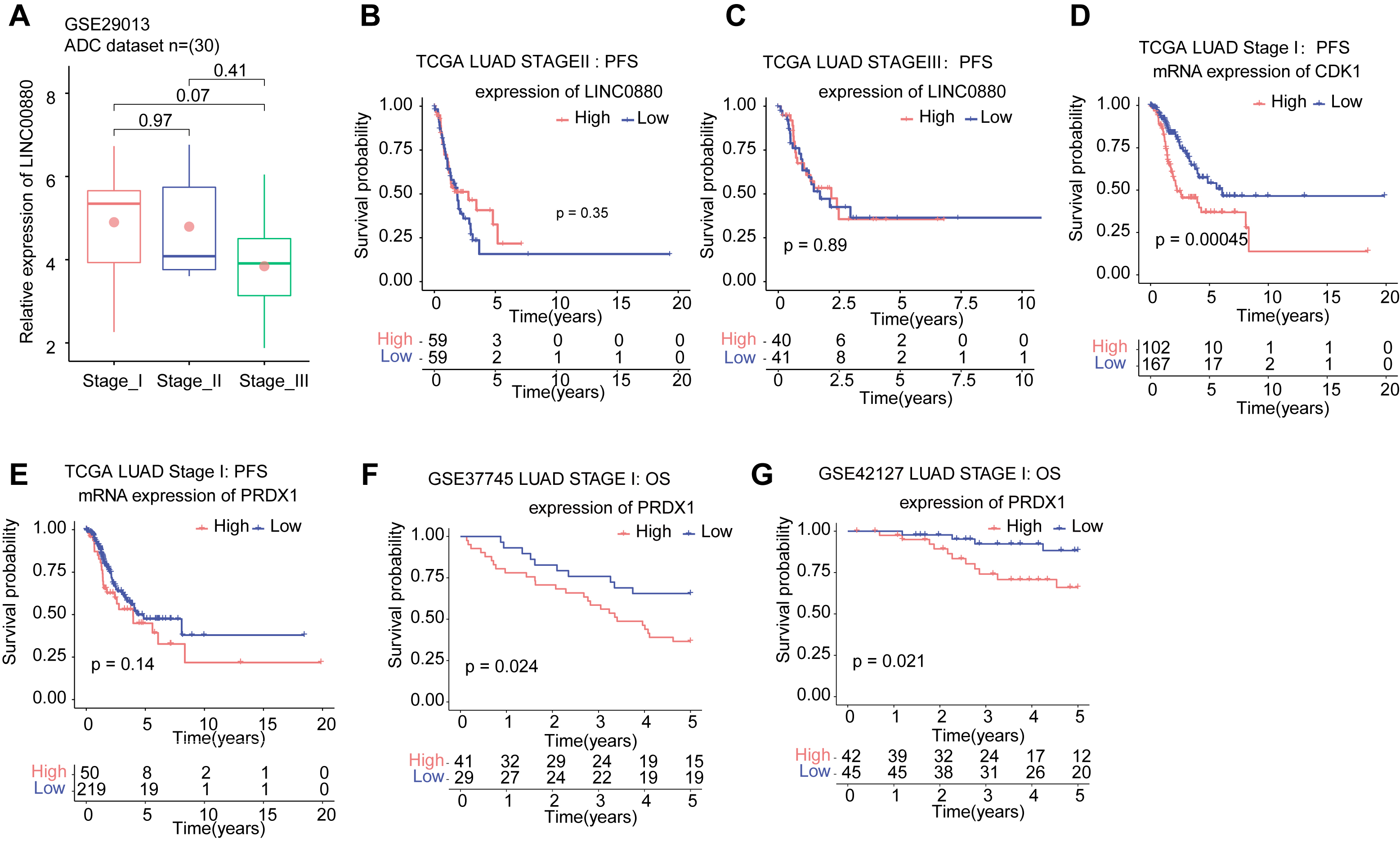

Supplement: Supplementary file 6 — Figure. S6 [file 41419_2023_6047_MOESM6_ESM.jpg]
