# Supplementary material for: The super-enhancer-driven lncRNA LINC00880 acts as a scaffold between CDK1 and PRDX1 to sustain the malignance of lung adenocarcinoma
Source: Cell Death Dis. 2023 Aug 24;14(8):551. doi: 10.1038/s41419-023-06047-w (PMC10449921; doi:10.1038/s41419-023-06047-w)
Supplement: Supplementary file 8 — Supplementary-Table [file 41419_2023_6047_MOESM8_ESM.docx]

**Supplementary Tables**

**Supplementary Table 1. Relationships between LINC00880 expression and clinicopathological parameters of LUAD**

| Parameter | No. of patients | LINC00880(low) | LINC00880(high) | *P*-value |
| --- | --- | --- | --- | --- |
| **Age**(yr)  ＞60  ≤60  **Sex**  Male  Female  **Clinical stage**  Early stage (I)  Late stage (II-III)  **pT status**  T1-T2  T3-T4  **Lymph node**  **metastasis**  N0  N1-N3 | 81  71  83  69  59  93  129  33  68  84 | 30  35  34  31  19  46  49  16  25  40 | 51  36  49  38  40  47  80  17  43  44 | 0.1417  0.7421  **0.0299**  0.3212  0.1913 |

**Supplementary Table 2. Sequences for the primers and siRNA**

| **Primer name** | **Sequence** |
| --- | --- |
| LINC00880-F  LINC00880-R  FOXP3-F  FOXP3-R  CDK1-F  CDK1-R  STAT1-F  STAT1-R  STAT4-F  STAT4-R  Promoter-F  Promoter-R  Enhancer1-F  Enhancer1-R  Enhancer2-F  Enhancer2-R  Enhancer3-F  Enhancer3-R  Enhancer4-F  Enhancer4-R  Enhancer5-F  Enhancer5-R  U6-F  U6-R  ACTIN-F  ACTIN-R  GAPDH-F  GAPDH-R  5’-RACE-R1  5’-RACE -R2  3’-RACE -F1  3’-RACE -F2 | 5’-AATGAATGTGGGGCTCAGGG-3’  5’-GCCCAGTTTCCACATTGCTG-3’  5’- GTGGCCCGGATGTGAGAAG-3’  5’- GGAGCCCTTGTCGGATGATG-3’  5’- AAACTACAGGTCAAGTGGTAGCC-3’  5’- TCCTGCATAAGCACATCCTGA-3’  5’- GCGCGCAGAAAAGTTTCATTTGC-3  5’- CTGAGACATCCTGCCACCTTG-3  5’- CCTGACATTCCCAAAGACAAAGC-3  5’- TCTCTCAACACCGCATACACAC-3  5’- CCTCCGACTCCACCATCCTA-3’  5’- TACCCCGGTCCTACTCCTTG-3’  5’- AGGAAGACAGGTGTGCTGTT-3’  5’- ACGAGCAATCGAGATGTACCT-3’  5’- CTGGAGAATCGAGGCTCTGC-3’  5’- ATGAACTGTCCCTTCCCTGG-3’  5’- GAGAGGATGGATGCTCAGCC-3’  5’- TTCTGTTTCAGGGCTCAGGG-3’  5’- ACCTCTTCACGATCCAGGTT-3’  5’- CTGGGAGTCTTGACAGCCTC-3’  5’- TCAGTGTCTCCTTTTCCAGACT-3’  5’- GTCCTTCCTCCCCTTGTGTG-3’  5’- CTCGCTTCGGCAGCACA-3’  5’- AACGCTTCACGAATTTGCG-3’  5’-CATGTACGTTGCTATCCAGGC-3’  5’-CTCCTTAATGTCACGCACGAT-3’  5’-ACAGTTGCCATGTAGACC-3’  5’-TTTTTGGTTGAGCACAGG-3’  5’-GCCATGGCCTGACATCGAATGCCTAA-3’  5’-ATGCACTGGCGAGCTTCCTGGGGACAA-3’  5’-CCACTGAGACCCCAAAGGCGCATTC-3’  5’-GCAGGCAGAGGCAAGAGAAGAGGAAAG-3’ |

| **Oligo set**  si-CDK1-1  si-CDK1-2  si-CDK1-3  si-LINC00880-1  si-LINC00880-2  si-LINC00880-3  si-PRDX1-1  si-PRDX1-2 | **Sequence**  5’-GGAACTTCGTCATCCAAAT-3’  5’-GTACTGCAATTCGGGAAAT-3’  5’-GGTTATATCTCATCTTTGA-3’  5’-ATGAATTCTGGAGGACAAAATTC-3’  5’-CTCTATGCTAATTTTTTCCAAAA-3’  5’-ATCTTTTTAGCATCATAGACACT-3’  5’-GCTCAGGATTATGGAGTCTTA-3’  5’-ATGTGGAATAGAACAAACTTTAG-3’ |
| --- | --- |
| si-PRDX1-3  si-FOXP3-1  si-FOXP3-2  si-FOXP3-3  si-STAT1  si-STAT4 | 5’-AAGATTGTAAAGTCAAATTATTC-3’  5’-TCCGTTTAAGTCTCATAATCAAG-3’  5’-CTCATCGAAAAAAATTTGGATTA-3’  5’-GGCAAAAATAAATGAATTCATCA-3’  5’-CTGGAAGATTTACAAGATGAATT-3’  5’-GCGAGACTACAAAGUUATTATTT-3’ |

**Supplementary Table 3. The antibodies used in this study**

| **Protein Name** | **Company** | **Catalog Number** | **Application** |
| --- | --- | --- | --- |
| FOXP3  STAT1  STAT4  CDK1  P-CDK1(T161)  p-CDK1(T14)  p-CDK1(Y15)  ACTIN  HSP90  cyclin B  CDK7  MNAT1  cyclin H  PRDX1  p-PRDX1  PTEN  AKT  p-AKT  FLAG  MYC | Proteintech  Cell Signaling Technology  Cell Signaling Technology  Cell Signaling Technology  Abcam  Affinity  Cell Signaling Technology  Cell Signaling Technology  Cell Signaling Technology  Cell Signaling Technology  Cell Signaling Technology  Abcam  Abcam  Abcam  Cell Signaling Technology  Cell Signaling Technology  Cell Signaling Technology  Cell Signaling Technology  Cell Signaling Technology  Proteintech | 22228-1-AP  #14994  #2653  #9116  ab201008  DF2944  #4539  #3700  #4877  #12231  #2916  ab154802  ab92376  ab109506  #14041  #9188  #9272  #4060  #8146  60003-2-lg | WB (1:1000), ChIP (1:50), IHC (1:200)  ChIP (1:50)  ChIP (1:50)  WB (1:1000), IP (1:200)  WB (1:1000), IHC (1:200)  WB (1:1000)  WB (1:1000)  WB (1:1000)  WB (1:1000)  WB (1:1000)  WB (1:1000)  WB (1:1000)  WB (1:1000)  WB (1:1000), IP (1:200)  WB (1:1000), IHC (1:200)  WB (1:1000)  WB (1:1000)  WB (1:1000), IHC (1:200)  WB (1:1000), IP (1:200)  WB (1:1000), IP (1:200) |

**Supplementary Table 4. Sequence of enhancers**

|  | Sequence | Location |
| --- | --- | --- |
| **E1** | tattcctttatactcagtcctagtttgttttagatctgggcctctgggcccatt  aatttgtataggatgataatttgtataggaagacaggtgtgctgtttttcttc  aatccttgaggatattaggaaaaactttaaccaattaagggatcccatcac  ctgacgtttctttggggaaaatgacttttgaaggcatgtggggattgtcaa  ggaaataattttccctttaaattacatgtttgtacgtaagaggaatgctgag  aggatatcaagaggcagtggtcactcatggcactattttcaaagttagtta  aacaaggaaaccaactcacatttccccagtttagcttttcatttttaaaacttg  ctgaattcaggtacatctcgattgctcgtggtaataaaggag | chr3:157081203-157081611 |
| **E2** | caatttcctggagaatcgaggctctgcctcagatgttcggagcctccgca ggatgaacacctccatttcaattccaggggctttgatcaacctgtgcctcc  cttgatccccaacttgtgggcaccttcccttgagcagtgcccccacgagg  tacacctttcccgggaaacaaaaatcattccttctttgggtcctgcaagct  gtaaagaaccaatacagcactgtgctactgaacatgttgccactatagaa  gccccttgtgttttgaagagtacatctctatagccattcgtttattcatcaga  aaccagctagaagccaggccctgtgctgggcgctggagacatggaag  aaactgggctgaggctcccgccttgaagagcttgtggcccagggaagg gacagttcatctgtcattgattgccacgtga | chr3:157082014-157082444 |
| **E3** | gttcttccaaactcttgacttttggaaaaaattagcatagagaggatggatg  ctcagccatggcctgacatcgaatgcctaatgcagaggtgaagacacctg  cacagtcccatgcactggcgagcttcctggggacaagggcagtgccttga  tcacctctcttcctcgtgcagtaaatgtttgttggatgaatggttgtcaactcta  gtcctgtcagtcctccctgagccctgaaacagaaattaggaaataataacaa  ttagcaggtcctatactcctagatttaagatttttgaacaaataagggaaaatt  at | chr3:157083046-157083360 |
| **E4** | aaatgtaatctcttgacagaatatctaaaaagacagattattgattttaatctc  tcaggagacctaacctcttcacgatccaggtttcctgattttcagacctgagt  taagttgctatggatacagagtgacaggccagtgaaggaaactgggga  cagggagtggcctgcccacaggtgagatatactgtctttaagcttgttagg  gatagaaaagggggctagcctagaattgtcctctgggacataaggtaat  ggtcctgctcccactttttaaacagtggtttgaaagttttctcagggtgtgca  tacatttggacgtacaagttctgctacgttttaaaaagcaaaacagaataa  acaaacatatatgaaaacaaacaataataatgataatctggggctttcctca  aacttggctggaagttactggcacacaaatagtagaggtgtttgggggtt  ttctgctatgaaggagtccttgatttgtactatctcaataaagcaaccttctttt  gacctgcctttggaagaagtaggtgaatttctaagagcccagctgtgctg  gaccctaggaggctgtcaagactcccagggtaggaatgttcctccacaa  ctttgaatttcaatttc | chr3:157086846-157087477 |
| **E5** | gctaaaaggtcatctatacaaatccttaagatggtgaacctctacaacctta  ctttaattttaggagatatttcatttcatgccttaagttttgcaattattttcagtg  tctccttttccagacttcttagaggacttggaaagaaaatgaaatgacgttg  aacgaaatcaaagtaaattgagtgatctgaaacgatttgatgtcagcaaga  attccccatacagctcacagccctaccccgagcacgcctccctcttgggtat  ggtgagttcagctccaaatttattctgacaagaaagcacaggcaatggctt  cagtgggtctaaatcaggtgaaaattcaaagcaatcacccttcctggaca  atcttctgctttctcagtgcttatcatggtctgaaaattcctatttaaagaagc  agagctttcttttaagtcctaacagaacaaagtcacactcaacagtgcaca  ggcacacgccgggatcgagaatgcatttttggcaaaaagtctcctcttttc  tctgtctcctccagctctctgcaggcccaaaccttgcagaagccattccatg  tttcctgagcaggaaaagacaaactcagaccgacaaccttggacttcaaa  agactgcacacaaggggaggaaggactgagagtaccaaggcgtgaga  attc | chr3:157087752-157088425 |

**Supplementary Table 6. List of top 50 LINC00880 binding proteins**

| \| **Rank** \| **Accession** \| **Gene** \| **Mw(kD)** \| **%Cov(95)** \| \| --- \| --- \| --- \| --- \| --- \| |
| --- | --- | --- | --- | --- | --- |
| \| 1 \| P07355 \| ANXA2 \| 38.604 \| 56.92999959 \| \| --- \| --- \| --- \| --- \| --- \| \| 2 \| Q9HCC0 \| MCCC2 \| 61.333 \| 46.93999946 \| \| 3 \| P05141 \| SLC25A5 \| 32.852 \| 43.14999998 \| \| 4 \| P63104 \| YWHAZ \| 27.745 \| 41.62999958 \| \| 5 \| P06493 \| CDK1 \| 34.095 \| 39.21000034 \| \| 6 \| Q06830 \| PRDX1 \| 22.11 \| 39.09999996 \| \| 7 \| P09525 \| ANXA4 \| 35.883 \| 38.17999929 \| \| 8 \| Q96RQ3 \| MCCC1 \| 80.473 \| 35.99999964 \| \| 9 \| P23528 \| CFL1 \| 18.502 \| 35.06000012 \| \| 10 \| P62826 \| RAN \| 24.423 \| 34.81000036 \| \| 11 \| P60174 \| TPI1 \| 30.791 \| 33.98999989 \| \| 12 \| P07900 \| HSP90AA1 \| 84.66 \| 33.66000026 \| \| 13 \| P62913 \| RPL11 \| 20.252 \| 33.4800002 \| \| 14 \| P62241 \| RPS8 \| 24.205 \| 33.45999986 \| \| 15 \| P62750 \| RPL23A \| 17.695 \| 32.81999946 \| \| 16 \| P62701 \| RPS4X \| 29.598 \| 32.54999936 \| \| 17 \| A0A494BZZ2 \| TPM1 \| 28.823 \| 31.94999963 \| \| 18 \| P23396 \| RPS3 \| 26.688 \| 31.93000004 \| \| 19 \| P68431 \| HIST1H3A \| 15.404 \| 31.76000014 \| \| 20 \| Q9UHB6 \| LIMA1 \| 85.226 \| 31.59000024 \| \| 21 \| Q8NC51 \| SERBP1 \| 44.965 \| 31.0299997 \| \| 22 \| P35268 \| RPL22 \| 14.787 \| 30.15999988 \| \| 23 \| P62753 \| RPS6 \| 28.681 \| 29.63900015 \| \| 24 \| P07195 \| LDHB \| 36.638 \| 29.58100036 \| \| 25 \| P0DMV8 \| HSPA1A \| 70.052 \| 29.516 \| \| 26 \| P52907 \| CAPZA1 \| 32.923 \| 29.09100026 \| \| 27 \| P35580 \| MYH10 \| 228.999 \| 28.95700008 \| \| 28 \| P06703 \| S100A6 \| 10.18 \| 28.88900012 \| \| 29 \| P83731 \| RPL24 \| 17.779 \| 28.28000009 \| \| 30 \| P12004 \| PCNA \| 28.769 \| 28.04599971 \| \| 31 \| P52597 \| HNRNPF \| 45.672 \| 27.9520002 \| \| 32 \| P62805 \| HIST1H4A \| 11.367 \| 27.76700005 \| \| 33 \| P61981 \| YWHAG \| 28.303 \| 27.69200027 \| \| 34 \| P46776 \| RPL27A \| 16.561 \| 27.43200034 \| \| 35 \| P31943 \| HNRNPH1 \| 49.229 \| 27.34999999 \| \| 36 \| P15121 \| AKR1B1 \| 35.853 \| 27.27799982 \| \| 37 \| P67936 \| TPM4 \| 28.522 \| 27.25800022 \| \| 38 \| P62269 \| RPS18 \| 17.719 \| 27.23700002 \| \| 39 \| P62899 \| RPL31 \| 14.463 \| 27.19999969 \| \| 40 \| P35232 \| PHB \| 29.804 \| 26.98499978 \| \| 41 \| P62280 \| RPS11 \| 18.431 \| 26.96199983 \| \| 42 \| Q07020 \| RPL18 \| 21.634 \| 26.91500008 \| \| 43 \| Q00325 \| SLC25A3 \| 40.095 \| 26.90599978 \| \| 44 \| Q15233 \| NONO \| 54.232 \| 26.79399967 \| \| 45 \| P62263 \| RPS14 \| 16.273 \| 26.73400015 \| \| 46 \| P60981 \| DSTN \| 18.506 \| 26.66700006 \| \| 47 \| P63244 \| RACK1 \| 35.077 \| 26.62499964 \| \| 48 \| P05198 \| EIF2S1 \| 36.112 \| 24.34900033 \| \| 49 \| Q16658 \| FSCN1 \| 54.53 \| 21.2880002 \| \| 50 \| P61254 \| RPL26 \| 17.258 \| 16.20700009 \| |
