# Supplementary material for: The super-enhancer-driven lncRNA LINC00880 acts as a scaffold between CDK1 and PRDX1 to sustain the malignance of lung adenocarcinoma
Source: Cell Death Dis. 2023 Aug 24;14(8):551. doi: 10.1038/s41419-023-06047-w (PMC10449921; doi:10.1038/s41419-023-06047-w)

Figure 4E CDK1


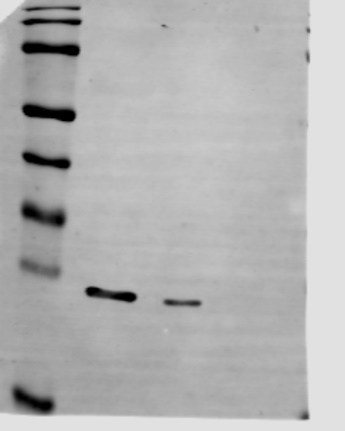


Figure 4E ACTIN


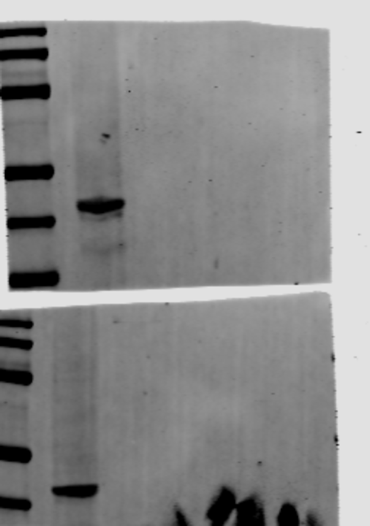


Figre 4F CDK1


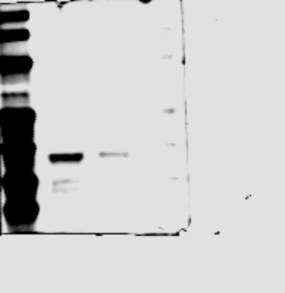


Figre 4F ACTIN


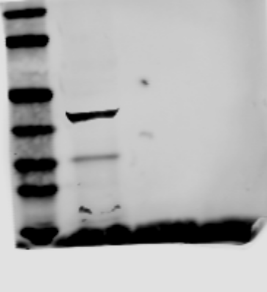


Figre 4H CDK1


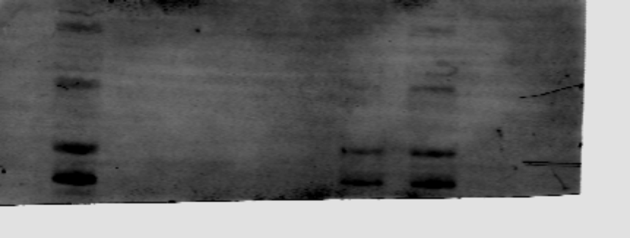


Figure 4G cyclinB


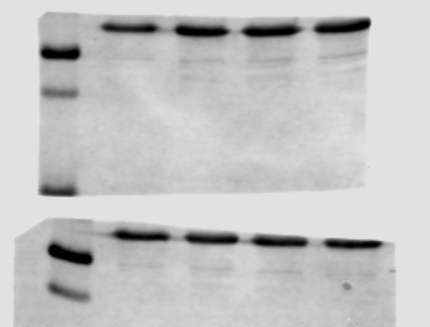


Figure 4G p-CDK1(T14)


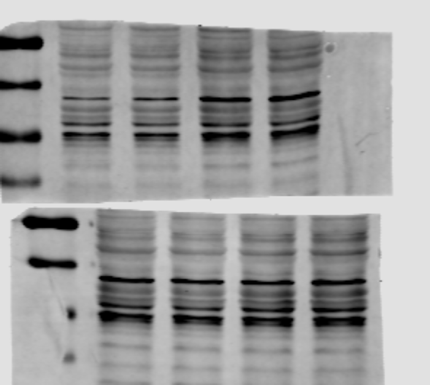


Figure 4G p-CDK1(Y15)


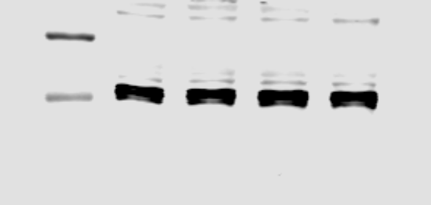


Figure 4G p-CDK1(T161)


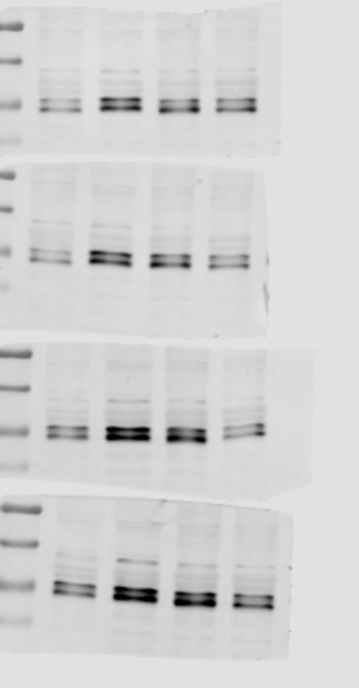


Figure 4G p-CDK1(HSP)


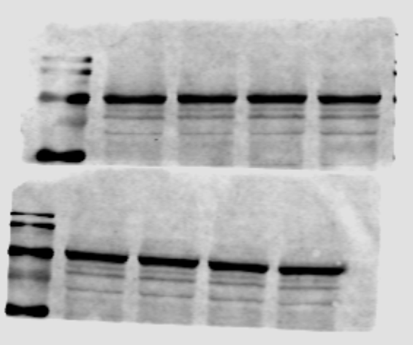


Figure 4K CDK7


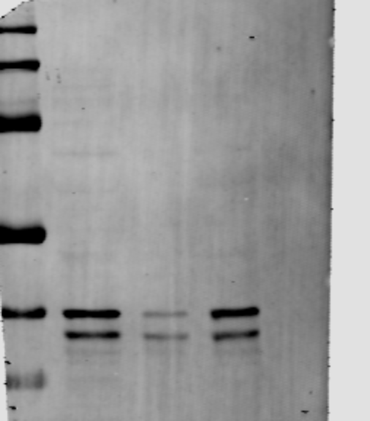


Figure 4K cyclin H


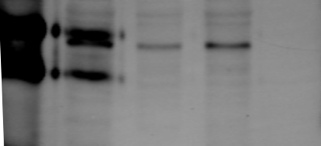


Figure 4K MNAT1


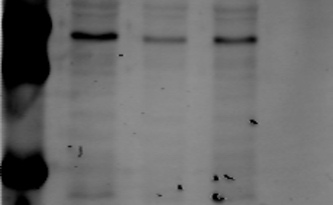


Figure 4K CDK1


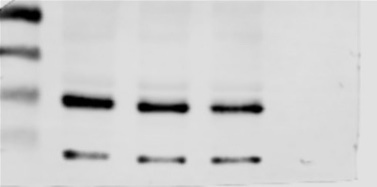


Figure 4M p-CDK1(T14)


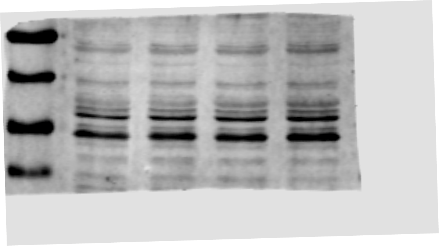


Figure 4M p-CDK1(Y15)


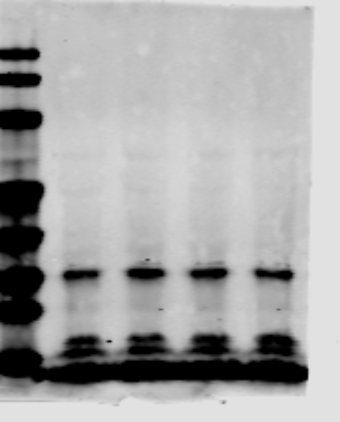


Figure 4M p-CDK1(T161)


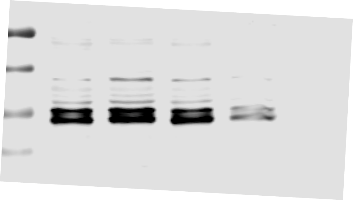


Figure 4M p-CDK1(HSP)


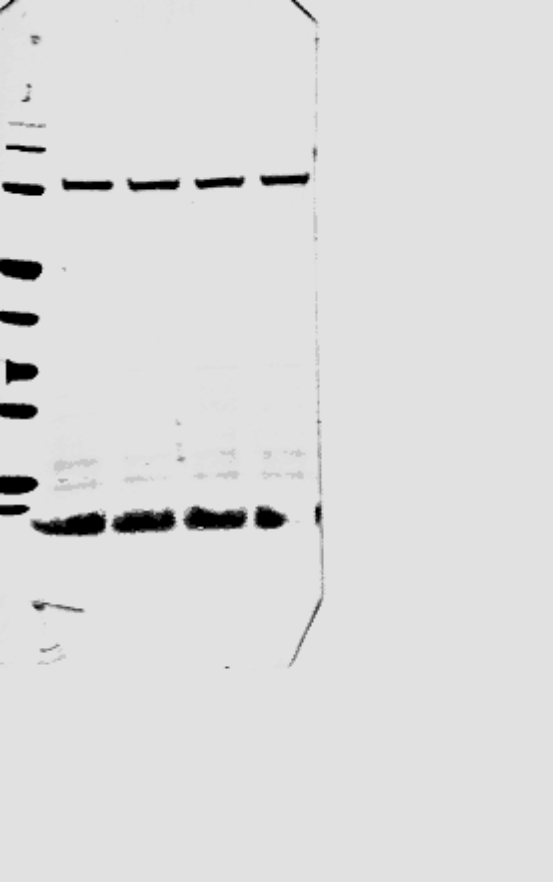


Figure 4N CDK7


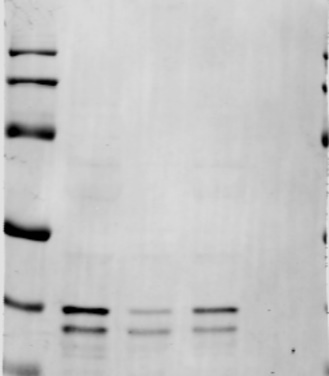


Figure 4N cyclin H


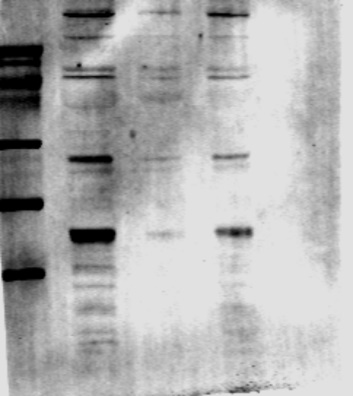


Figure 4N MNAT1


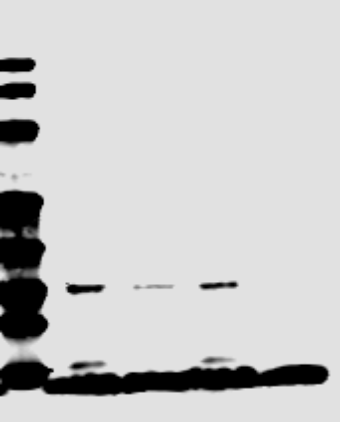


Figure 4N CDK1


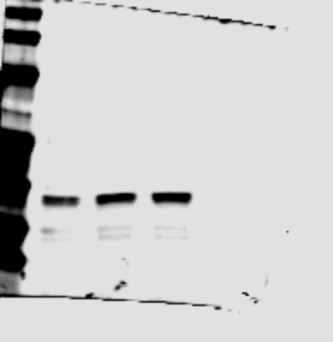


Figure 4N ACTIN


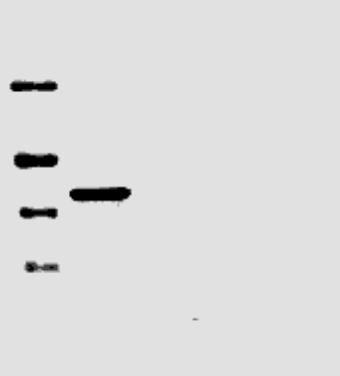


Figure 5B (up PRDX1)


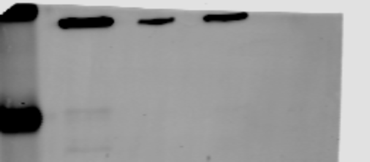


Figure 5B (up CDK1)


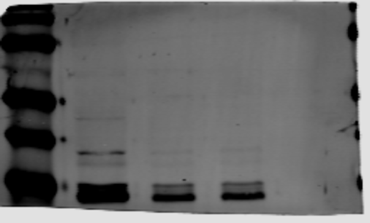


Figure 5B (up ACTIN)


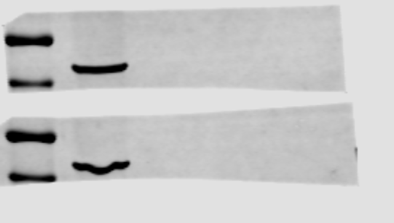


Figure 5B (dn CDK1)


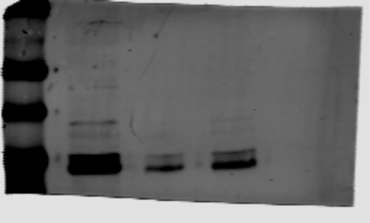


Figure 5B (dn PRDX1)


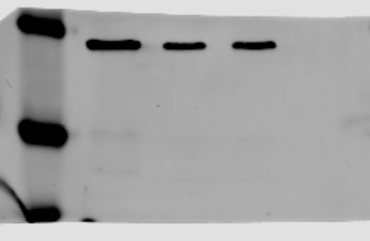


Figure 5B (dn ACTIN)


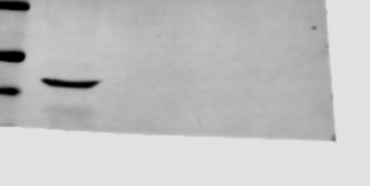


Figure 5C MYC-PRDX1(up)


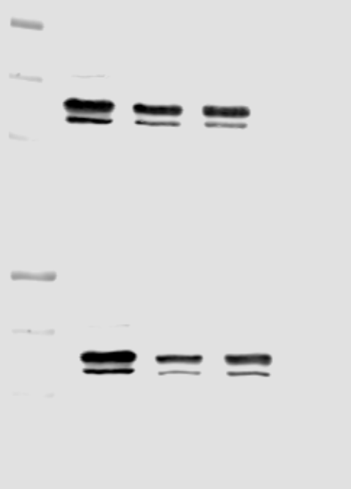


Figure 5C ACTIN（up）


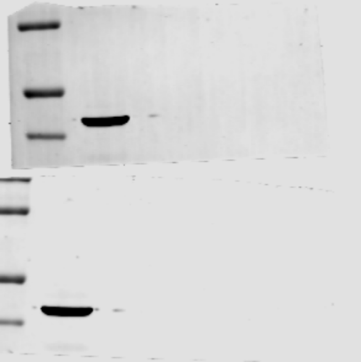


Figure 5C MYC-PRDX1(dn)


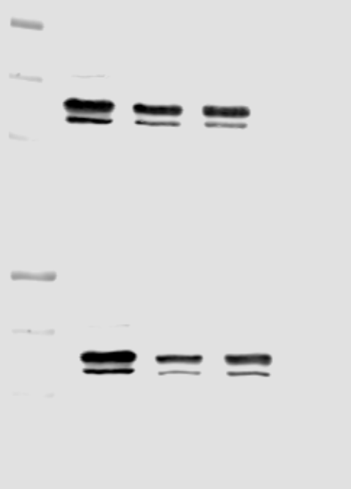


Figure 5C FLAG-CDK1(up)


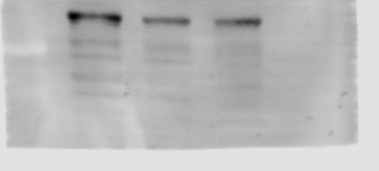


Figure 5C FLAG-CDK1(dn)


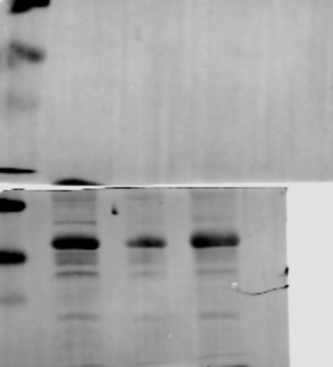


Figure 5C ACTIN（dn）


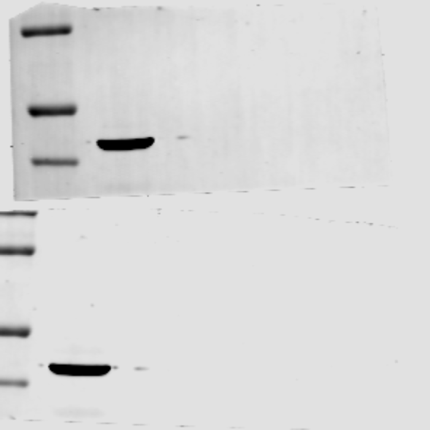


Figure 5E PRDX1


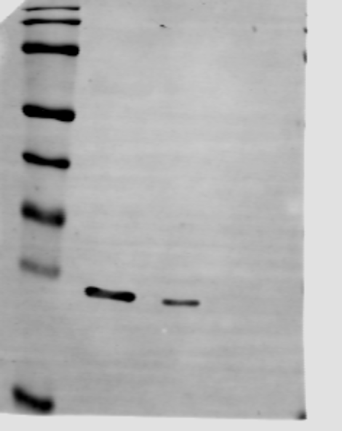


Figure 5E ACTIN


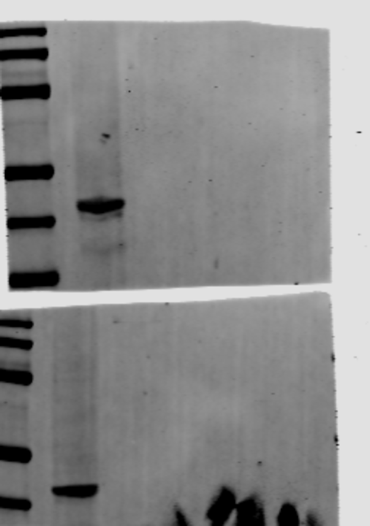


Figure 5G


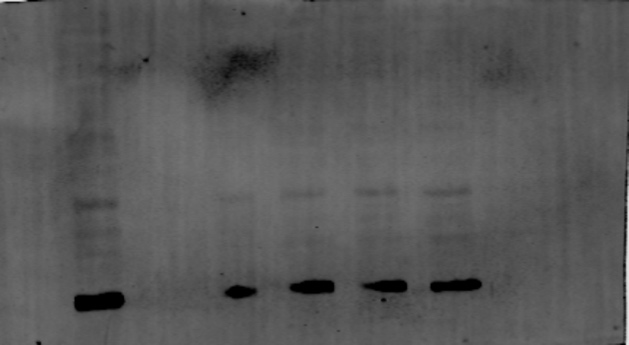


Figure 5H CDK1


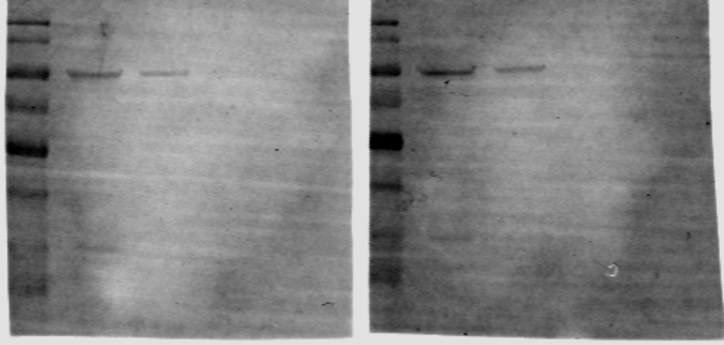


Figure 5H PRDX1


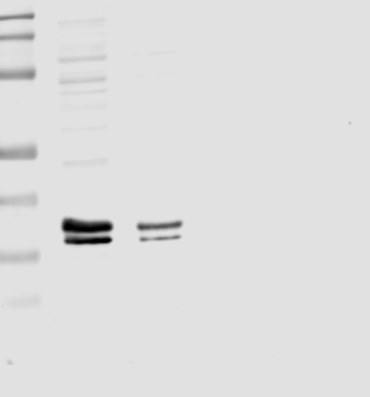


Figure 5H ACTIN


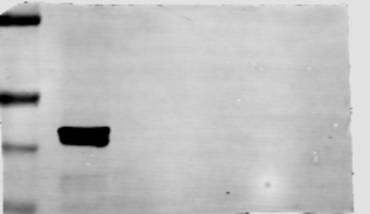


Figure 5I PRDX1


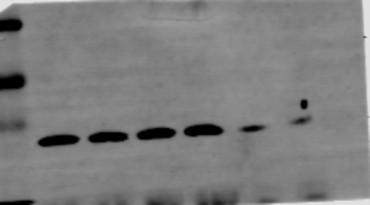


Figure 5I p-PRDX1


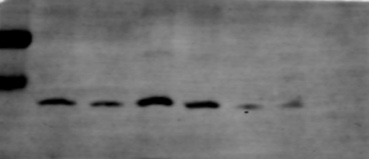


Figure 5I PTEN


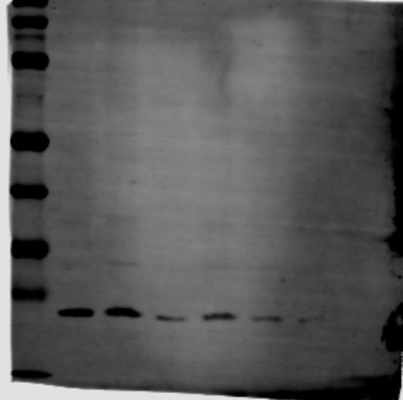


Figure 5I AKT


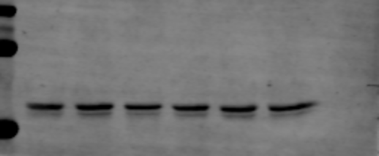


Figure 5I p-AKT


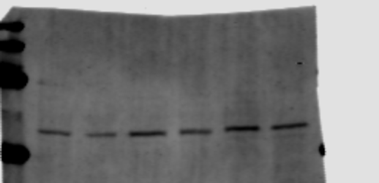


Figure 5I-ACTIN


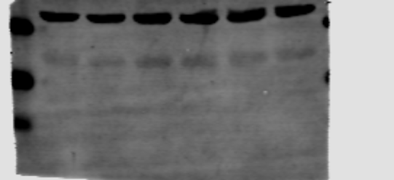


Figure 5j PRDX1


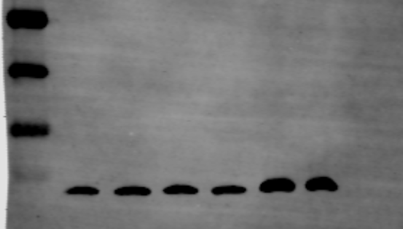


Figure 5j p-PRDX1


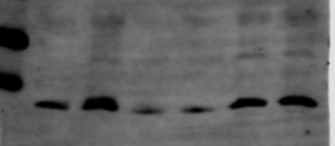


Figure 5j PTEN


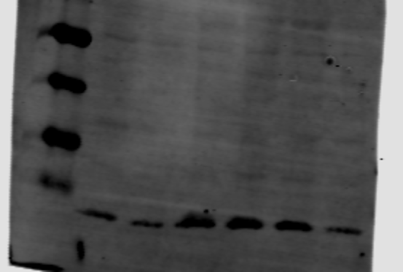


Figure 5j AKT


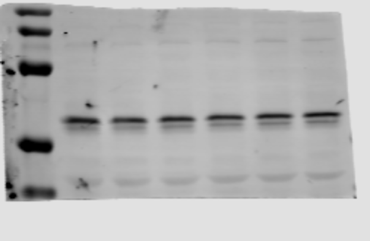


Figure 5j p-AKT


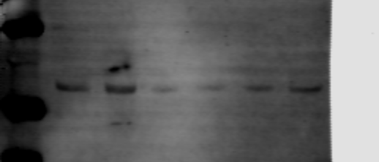


Figure 5j ACTIN


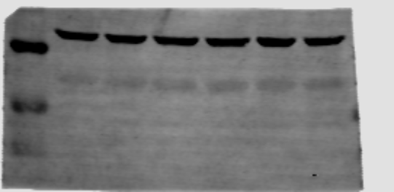


Figure S4B CDK1


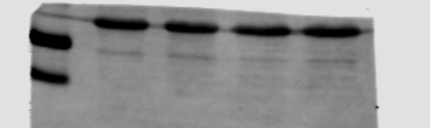


Figure S4B HSP90


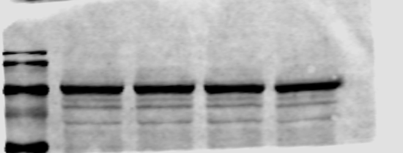


Figure S4C CDK7


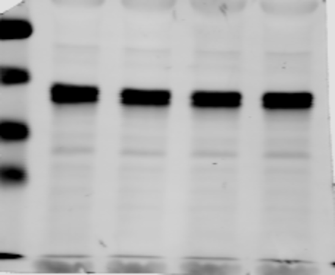


Figure S4C MNAT1


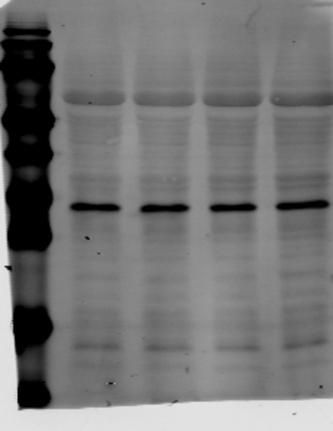


Figure S4C cyclin H


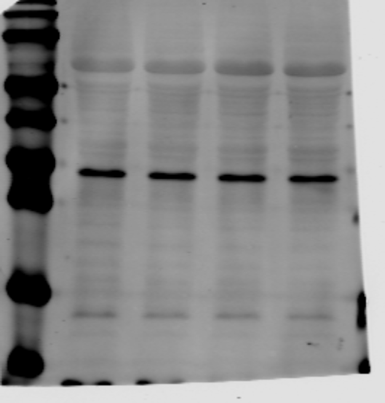


Figure S4C HSP90


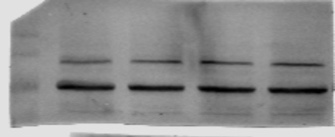


Figure S4E CDK1


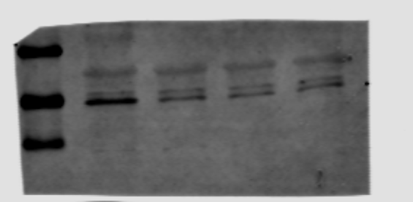


Figure S4E HSP90


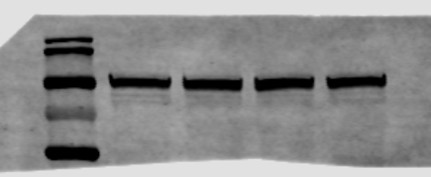


Figure S5C PRDX1


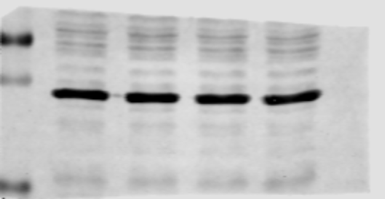


Figure S5C p-PDRX1


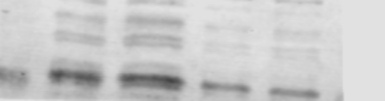


Figure S5C PTEN


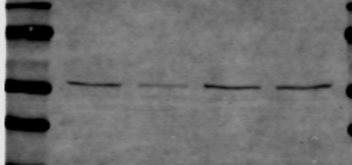


Figure S5C AKT


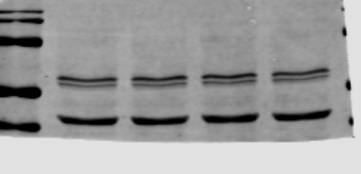


Figure S5C p-AKT


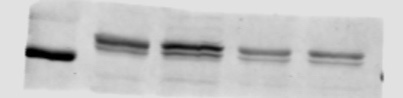


Figure S5C GAPDH


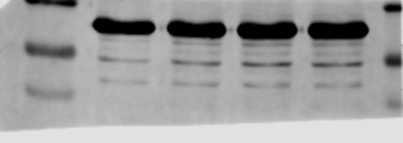

Supplement: Supplementary file 9 — western_blot_supplement [file 41419_2023_6047_MOESM9_ESM.docx]
