# Supplementary material for: The super-enhancer-driven lncRNA LINC00880 acts as a scaffold between CDK1 and PRDX1 to sustain the malignance of lung adenocarcinoma
Source: Cell Death Dis. 2023 Aug 24;14(8):551. doi: 10.1038/s41419-023-06047-w (PMC10449921; doi:10.1038/s41419-023-06047-w)
Supplement: Supplementary file 10 — Figure legends [file 41419_2023_6047_MOESM10_ESM.docx]

**Figure. S1.** (A) Flow chart of gene selection (B) Heatmap of top selected gene of microarray dataset (|Log2FC|>6 & adj.pvalue<0.05). (C) The pan-cancer expression analysis of LINC00880 in IMNLINC dataset. (D) Assessment of coding potential of LINC00880 by using phyloCSF. (E) The transcript of LINC00880 from RACE and NCBI. (F) Relative expression of LINC00880 detected by qRT-PCR in PC9 cells using random hexamer or oligo (dT)18 primers.

**Figure .S2.** (A) FOXP3 expression was positively correlated with LINC00880 expression. (Spearman’s correlation analysis). (B) ChIP analysis using antibodies against STAT1 and STAT4, Immunoglobulin G (IgG) was used as the negative control. (C) Detection of FOXP3 expression in PC9 cells treated with FOXP3 knockdown by q-PCR. (D) Detection of STAT1 expression in PC9 cells treated with STAT1 knockdown by q-PCR. (E) Detection of STAT4 expression in PC9 cells treated with STAT4 knockdown by q-PCR. (F) Detection of LINC00880 expression in PC9 cells treated with STAT1 knockdown by q-PCR. (G) Detection of LINC00880 expression in PC9 cells treated with STAT4 knockdown by q-PCR.

The data are shown as the mean ± S.D. of at least three replicates (*P < 0.05, **P < 0.01, ***P < 0.001).

**Figure. S3.** (A-B) qRT-PCR detection of LINC00880 expression in A549 and PC9 cells with LINC00880 overexpression or knockdown. The data are shown as the mean ± S.D. of at least three replicates (*P < 0.05, **P < 0.01, ***P < 0.001).

**Figure. S4.** (A-B) qRT-PCR and western blotting detection of CDK1 levels in PC9 cells with LINC00880 overexpression or knockdown. (C) Western blotting detection of CAKs levels in PC9 cells with LINC00880 overexpression or knockdown. (D-E) qRT-PCR and western blotting detection of CDK1 expression in PC9 cells with CDK1 knockdown. (F) Assessment of CDK1 kinase activity in PC9 cells treated with 20 nM RO-3306 for 24h. (G-H) Colony formation assays and CCK-8 assays showed that overexpression of CDK1 partly rescued cell proliferation impaired by LINC00880 knockdown, and that overexpression of LINC00880 cannot restore cell proliferation impaired by CDK1 knockdown or CDK1 inhibitor RO-3306. The data are shown as the mean ± S.D. of at least three replicates (*P < 0.05, **P < 0.01, ***P < 0.001).

**Figure. S5. (A)**The represent confocal images demonstrate the co-localization of CDK1 and PRDX1 in PC9 cells**.** (B) Veen diagram show the number of overlap protein among three groups. (C) Western blotting showed that LINC00880 regulated PTEN/AKT pathway relying on the kinase activity of CDK1. (D) CCK-8 assays showing PRDX1 knockdown or using of PTEN inhibitor SF1670 partly rescued cell proliferation impaired by LINC00880 knockdown. The data are shown as the mean ± S.D. of at least three replicates (*P < 0.05, **P < 0.01, ***P < 0.001).

**Figure. S6.** (A)LINC00880 expression analysis in GSE29013 (LUAD dataset, n=30). (B-C) Survival analysis of stage II/III LUAD patients with low or high LINC00880 expression. (D-E) TCGA-LUAD PFS survival analysis in stage I patients according to the mRNA expression of CDK1 and PRDX1. (F-G) GSE37745 and GSE42127 LUAD survival analysis in stage I patients according to the expression of PRDX1.x
